# Zebrafish as model system for the biological characterization of CK1 inhibitors

**DOI:** 10.3389/fphar.2023.1245246

**Published:** 2023-09-11

**Authors:** Laura Meier, Bernd Martin Gahr, Aileen Roth, Adrian Gihring, Stefan Kirschner, Clemens Woitaske-Proske, Joana Baier, Christian Peifer, Steffen Just, Uwe Knippschild

**Affiliations:** ^1^ Surgery Center, Department of General- and Visceral Surgery, University Hospital Ulm, Ulm, Germany; ^2^ Molecular Cardiology, Department of Internal Medicine II, University Hospital Ulm, Ulm, Germany; ^3^ Institute of Pharmacy, Christian-Albrechts-University of Kiel, Kiel, Germany

**Keywords:** CK1delta, CK1 specific small molecule inhibitors, zebrafish, CK1 orthologues, heart failure, blood stasis

## Abstract

**Introduction:** The CK1 family is involved in a variety of physiological processes by regulating different signaling pathways, including the Wnt/β-catenin, the Hedgehog and the p53 signaling pathways. Mutations or dysregulation of kinases in general and of CK1 in particular are known to promote the development of cancer, neurodegenerative diseases and inflammation. There is increasing evidence that CK1 isoform specific small molecule inhibitors, including CK1δ- and CK1ε-specific inhibitors of Wnt production (IWP)-based small molecules with structural similarity to benzimidazole compounds, have promising therapeutic potential.

**Methods:** In this study, we investigated the suitability of the zebrafish model system for the evaluation of such CK1 inhibitors. To this end, the kinetic parameters of human CK1 isoforms were compared with those of zebrafish orthologues. Furthermore, the effects of selective CK1δ inhibition during zebrafish embryonic development were analyzed *in vivo*.

**Results:** The results revealed that zebrafish CK1δA and CK1δB were inhibited as effectively as human CK1δ by compounds G2-2 with IC_50_ values of 345 and 270 nM for CK1δA and CK1δB versus 503 nM for human CK1δ and G2-3 exhibiting IC_50_ values of 514 and 561 nM for zebrafish CK1δA and B, and 562 nM for human CK1δ. Furthermore, the effects of selective CK1δ inhibition on zebrafish embryonic development *in vivo* revealed phenotypic abnormalities indicative of downregulation of CK1δ. Treatment of zebrafish embryos with selected inhibitors resulted in marked phenotypic changes including blood stasis, heart failure, and tail malformations.

**Conclusion:** The results suggest that the zebrafish is a suitable *in vivo* assay model system for initial studies of the biological relevance of CK1δ inhibition.

## 1 Introduction

CK1 (formerly named casein kinase 1) is a serine/threonine-specific protein kinase family that is ubiquitously expressed in eukaryotes (Knippschild et al., 2014; Xu et al., 2019). In humans, six different CK1 isoforms (α, γ1-3, δ, and ε) and their different splice variants have been identified. While there is remarkably high amino acid conservation within the kinase domain of CK1 family members, their amino acid sequences differ considerably in their respective N- and C-terminal domains (Knippschild et al., 2005; Knippschild et al., 2014). The CK1 family plays an important role in the regulation of several signaling pathways, including Wnt/β-catenin (Sakanaka et al., 1999; Cruciat, 2014), Hedgehog (Price and Kalderon, 2002; Chen et al., 2011), and p53-dependent signaling (Knippschild et al., 1997; Dumaz et al., 1999; Venerando et al., 2010), and is therefore involved in the regulation of numerous cellular processes. These include embryonic development, apoptosis, DNA repair, proliferation, circadian rhythm and chromosome segregation (Knippschild et al., 2014; Schittek and Sinnberg, 2014; Fulcher and Sapkota, 2020; Francisco and Virshup, 2022; Roth et al., 2022). Mutations and dysregulated expression and/or activity of CK1 isoforms can promote the development of pathological conditions such as cancer (Tsai et al., 2007; Umar et al., 2007; Brockschmidt et al., 2008; Knippschild et al., 2014; Janovská et al., 2020), neurodegenerative diseases (Okochi et al., 2000; Kametani et al., 2009), and inflammatory processes (Marin et al., 2002; Wang et al., 2014).

Targeting CK1 with isoform-specific and potent small molecule inhibitors (SMIs) is a promising but challenging approach to provide novel therapeutics. CKI-7 (N-(2-amino-ethyl)-5-chloroisoquinoline-8-sulfonamide), was the first, albeit very nonselective, ATP-competitive CK1 targeting inhibitor introduced in 1989 (Chijiwa et al., 1989). Since then, great efforts have been made to develop highly specific and efficient CK1 isoform-specific inhibitors (Li et al., 2021). Several benzimidazole-based inhibitors from the linear type such as SR-3029, SR-2890, Bischof-5 and Bischof-6 showed highly specific inhibitory effects against CK1δ with 50% inhibitory concentration (IC_50_) values in the double-digit nanomolar range (Bischof et al., 2012; Bibian et al., 2013). Interestingly, compared to these compounds, the structurally similar linear inhibitors of Wnt production (IWPs), that were recently presented, also showed potent *in vitro* (and partially also *in vivo*) inhibition on CK1δ and ε (García-Reyes et al., 2018; Liu et al., 2019). Recently, Umbralisib (UKONIQ™), which primarily targets phosphatidylinositol 3-kinase (PI3K) delta but also shows a significant inhibition of CK1ε, was approved by the Food and Drug Administration (FDA) for the treatment of hematological malignancies (Dhillon and Keam, 2021). Consistent with this notion, triazolo [1,5-c]quinazoline 1 has been described as a derivate that acts as an osteogenic bone morphogenetic protein (BMP) signal enhancer through reciprocal inhibition of CK1 and PI3K isoforms (Wesseler et al., 2022). Furthermore, new CK1δ and ε inhibitors and the first highly selective CK1α inhibitor have been described recently (Němec et al., 2023). These examples clearly demonstrate that members of the CK1 family are important drug targets.

The development of SMIs is a multi-step process involving the determination of a variety of parameters such as potency, selectivity and efficiency of the compounds under standardized assay conditions to ensure comparability (Roth et al., 2021) before they can be tested in novel *in vitro* cell-based systems. Subsequently, the biological activity of SMIs has to be examined in more complex naturalistic model systems. Selected highly specific and potent inhibitors will therefore be further investigated in specific disease-associated or developmental animal models *in vivo* to demonstrate their efficacy against the target of interest. In addition, important data, including pharmacokinetics and toxicology, are derived from such *in vivo* models. Therefore, under strict ethical guidelines, the determination of half maximal effective concentration (EC_50_) values in *in vivo* models is inevitable. Although mice are still most commonly used for animal models, efforts are being made to establish alternatives due to the strict regulation of animal testing. In this sense, the early zebrafish model is also becoming increasingly popular in drug discovery (Keßler et al., 2015; Robinson et al., 2019).

The tropical zebrafish offers great advantages such as small body size, large reproductive capacity, genetic accessibility, and embryo transparency that allows visual assessment of embryogenesis and organogenesis (Penberthy et al., 2002; Yuan and Sun, 2009; Paone et al., 2018; Pott et al., 2020). Furthermore, 70% of all human genes and 82% of disease-associated human genes are found in the zebrafish genome (Howe et al., 2013; Bradford et al., 2017). Moreover, the zebrafish model can be used as alternative model, allowing reduction of necessary mouse models, since zebrafish embryos younger than 5 days after fertilization have not yet developed pain and distress perception and therefore can be used under the 3Rs principles (replacement, reduction and refinement of animal experiments) (Strähle et al., 2012). As for CK1, amino acid sequence alignment of the human and zebrafish CK1 isoforms α, δ and ε revealed great homology within their respective kinase domains, although there is a distinct variability in the C-terminal regions. Taken together, the highly similar features within the active site suggest comparable SMI binding and inhibition properties for the human and zebrafish CK1 isoforms. To evaluate the suitability of the zebrafish animal model for CK1-inhibition assays, we first determined the kinetic parameters of zebrafish and human CK1 isoforms. Next, we compared the effects of selected human CK1δ-specific SMIs on the inhibition of both, human and zebrafish CK1 isoforms. With regard to their ability to inhibit substrate phosphorylation, an initial screening of selected compounds revealed no major differences of IC_50_ values between the human and zebrafish CK1 isoforms. Because all CK1 isoforms are continuously expressed during zebrafish embryogenesis (Albornoz et al., 2007; Howe et al., 2013), we analyzed the effects of CK1-inhibition on embryonic development of zebrafish. Overall, the phenotype induced by CK1-SMI treatment is consistent with the developmental impairments following CK1δ-downregulation with morpholinos. Specific outcomes include cardiovascular dysfunction, malformation of the tail, necrosis, and early embryonic mortality. The observed effects on embryonic development and heart function, such as blood stasis, heart failure, tail malformation, necrosis and death were developed in a dose-dependent manner. Of note, among the CK1 specific SMIs used, G2-2 and G2-3 appeared to be most effective. This observation was confirmed by IC_50_ values for G2-2 of 345 nM against CK1δA, and 270 nM against CK1δB. Accordingly, treatment with G2-3 resulted in IC_50_ values of 514 nM against CK1δA and 561 nM against CK1δB. In conclusion, we provide strong evidence that zebrafish CK1δA and B are inhibited by SMIs as effectively as their human CK1 counterparts. In the zebrafish assay, inhibition of CK1δA and B by CK1δ-SMIs correlates with significant morphologic effects. Therefore, the early zebrafish model can be considered as a suitable *in vitro*/*in vivo* vertebrate animal model for initial studies of CK1δ inhibition.

## 2 Materials and methods

### 2.1 Generation of expression vectors

The prokaryotic expression vector pET28a (+) was synthesized from Biomatik (Toronto, ON, Canada). DNA fragments encoding for zebrafish (*Danio rerio - Dr*) CK1 isoforms δA, δB and ε were amplified by PCR (see primers in Supplementary Table S1) and inserted into pET28a (+) via Gibson Assembly (New England Biolabs Inc., United States). Successful introduction was confirmed by Sanger DNA sequencing (Eurofins Genomics, Munich, Germany).

### 2.2 Expression and purification of 6xHis-tagged Dr CK1 isoforms

Expression of recombinant 6xHis-DrCK1 isoforms was induced by adding 0.5 µM IPTG to an overnight *E. coli* Rosetta™ 2 (DE3) culture with an OD600 of 0.6–0.8 AU. Protein overexpression was conducted at 18°C and 120 rpm for 18 h. The culture was harvested by centrifugation and the bacteria pellets were stored at −80°C until further utilization. Bacteria were lysed using 6xHis lysis buffer, containing 50 mM sodium phosphate buffer (pH 7.0), 350 mM NaCl, 15 mM imidazole, 0.5% [v/v] NP-40, 10% [v/v] glycerine, 1 mM benzamidine, 1 mM aprotinin. TALON^®^ Metal Affinity Resin was added to the cleared lysate and after binding for 2 h at 4°C while rotating the beads were washed three times using a washing buffer, which is composed of 50 mM sodium phosphate buffer (pH 7.0), 350 mM NaCl, 15 mM imidazole, 10% [v/v] glycerine and 1 mM aprotinin. The recombinant proteins were eluted by adding elution buffer, containing 50 mM sodium phosphate buffer (pH 7.0), 300 mM imidazole and 1 mM aprotinin. Eluted proteins were dialyzed against imidazole-free sodium phosphate buffer (pH 7.0) two times for 30 min, aliquoted, shock frozen and stored at −80°C.

### 2.3 Expression and purification of GST-tagged human CK1δ

Expression and purification of GST-tagged human CK1δ and CK1ε was performed as described previously by Roth et al. (2021). In brief, the expression was induced by addition of 1 mM IPTG to an overnight *E. coli* Rosetta™ 2 (DE3) culture with an OD600 of 0.6–0.8 AU. After an expression period of 18 h at 15°C, the culture was harvested by centrifugation and the pellets were stored at −80°C until further use. Cell lysis was conducted by applying GST lysis buffer (20 mM Tris-HCl (pH 7.6), 150 mM NaCl, 10% [v/v] glycerol, 0.5% [v/v] NP40, 1 mM EDTA, 1 mM EGTA, 1 mM benzamidine, 1 mM aprotinin, 1 mM DTT) for 30 min on ice followed by ultrasonication of the lysate (Thermo Fisher Scientific Inc., Waltham, MA, United States) to fragment bacterial DNA. The cleared and filtered supernatant was loaded onto a GSTrap FF 1 mL column (Cytiva, Freiburg, Germany). The flow rate of the automated FPLC system (EttanLC, GE Healthcare, Chalfont St Giles, GB) was adjusted to 0.5 mL/min. After adequate column washing, the bound protein was eluted using elution buffer (50 mM Tris-HCl (pH 7.6), 5 mM reduced glutathione, 1 mM EDTA). Dialysis was performed three times for 10 min using glutathione-free elution buffer.

### 2.4 *In Vitro* kinase assay

Each *in vitro* kinase reaction was performed in a total volume of 15 µL containing 25 mM Tris-HCl (pH 7.0), 10 mM MgCl_2_, 100 µM EDTA, 2.8 µM of the substrate α-casein and 0.4 pmol [γ-^32^P]-ATP at 30°C. The kinase, as well as ATP-concentrations were used accordingly to the established standard conditions, which were determined following the workflow suggested by Roth et al. (2021). Reactions were stopped by adding 3 µL of 5x SDS loading buffer and incubating at 95°C for 5 min. Proteins were separated by SDS-PAGE and stained with Coomassie Brilliant Blue R250 (Waldeck GmbH & Co. KG, Muenster, Germany). Autoradiography visualized the incorporation of radioactively labelled γ-phosphate into the proteins. A quantitative analysis of the radioactive signal was performed by excising the proteins from dried gels and measuring phosphate incorporation via Cherenkov counting (Beckmann Scintillation Counter). For the determination of the initial velocity region, a linear regression was performed. Thereby, the number of included data-points was decreased stepwise, and the point of time with the highest maximum coefficient of determination (*R* ^2^) was chosen as the kinase-specific standard reaction time. For determination of the maximal velocity (V_max_) and Michaelis constant (K_m_) of ATP, various ATP concentrations in a range from 0.5 to 250 µM were tested. Raw data was used for the calculation of enzyme velocity (V) in [pmol/min]. Then, V was plotted over substrate (ATP) concentration and fitted to the Michaelis-Menten model via GraphPad Prism 8 (GraphPad Software, La Jolla, CA, United States). According to the Michaelis-Menten curve, K_m_, which is the concentration of the substrate, that is needed to obtain half V_max_, was calculated. For determination of IC_50_ values, the scintillation data measured in cpm was transformed to pmol of transferred phosphate. The data was then transformed logarithmically (X = log(X)), normalized to DMSO (100%) and zero (0%) before fitting to sigmoidal dose-response curves with variable slope with nonlinear regression using GraphPad Prism 7.

### 2.5 Small molecule inhibitors (SMIs)

The SMIs used in this study (summarized in Supplementary Table S2) were selected based on their inhibitory capacity toward CK1δ *in vitro*, whereby a broad range of inhibitors with varying efficiency were included. The determination of IC_50_-values was conducted prior to the standardization workflow establishment. Zebrafish embryos treated with 20 µM of the respective compound were phenotypically analyzed.

#### 2.5.1 Synthesis of compounds

##### 2.5.1.1 Synthesis of G1-4 and G1-5

**FIGURE 1:**
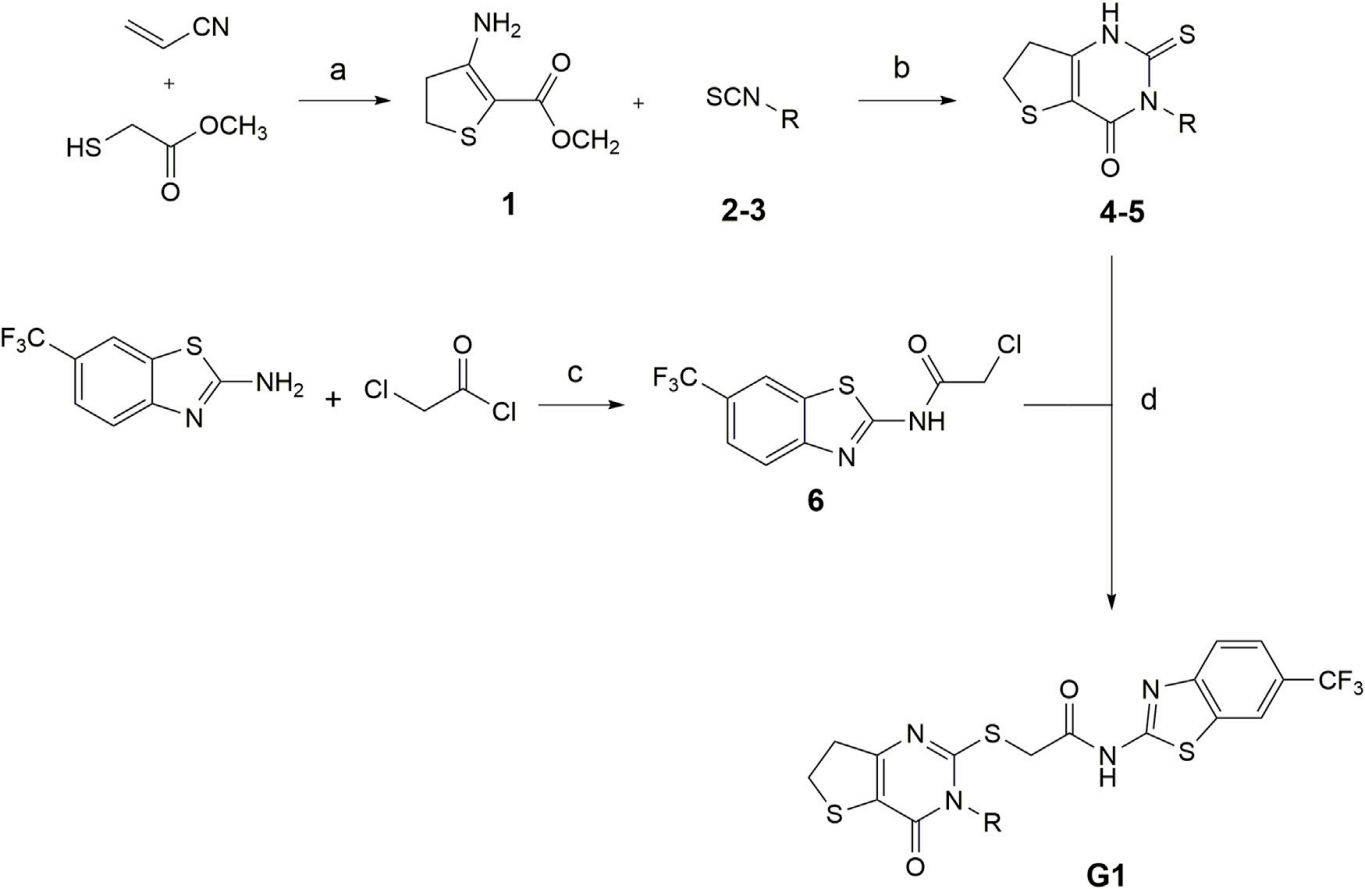
Conditions for the synthesis of compounds G1-4 and G1-5. (a) methanol, DBU 0°C–80°C, 24 h (b) Pyridine, 120°C, 24 h (c) TEA, DCM, rt, 24 h (d) DMF, TEA, N_2_, 80°C, 2 h (2,4G1-4: R = trifluoromethyl benzyl; 3,5G1-5: R = isobutyl. Modified after (Davoodnia et al., 2008).

The synthesis of compounds G1-4 and G1-5 was carried out as described by Wang et al., with alterations in the formation of the 6,7-dihydrothieno[3,2-d]pyrimidin-4(3H)-one scaffold (Wang et al., 2013). Here, we developed a synthesis route (see Figure 1), starting from the respective isothiocyanate derivates, as described by Davoodnia et al. (2008).

###### 2.5.1.1.1 Methyl-3-amino-4,5-dihydrothiophen-2-carboxylat (1)

The synthesis was carried out under nitrogen atmosphere. At 0°C methyl thioglycolate (4.20 mL, 46.7 mmol) was given to a solution of DBU (10.0 mL, 67.0 mmol) in abs. methanol (25 mL). Acryl nitrile (3.30 mL, 49.8 mmol) was added dropwise. The reaction was stirred for 5 h at 0°C, then for 16 h at 80°C. After cooling down, the solvent was removed at reduced pressure. The residue was suspended in saturated ammonium chloride solution and extracted with ethyl acetate (3 × 100 mL). The organic phase was dried over sodium sulfate and the solvent was removed under reduced pressure. The raw product was purified by flash column chromatography on silica gel (30% EA/PE). The product was obtained as a brown solid. Yield: 3.20 g (20.1 mmol; 43%). C_6_H_9_NO_2_S (M_*r*_ 159.21). ^1^H NMR (DMSO-*d*_6_): *δ* = 7.08 (s, 2 H, N*H*_2_), 3.57 (s, 3 H, C*H*_3_), 2.96-2.91 (m, 2 H, SCH_2_C*H*_2_), 2.85-2.78 (m, 2 H, SC*H*_2_CH_2_) ppm. ^13^C NMR (DMSO-d_6_): *δ* = 165.1 (*C*=O), 159.2 (*C*-NH_2_), 85.9 (S*C*C = O), 50.3 (*C*H_3_), 38.4 (S*C*H_2_CH_2_), 26.9 (SCH_2_ *C*H_2_) ppm. MS (ESI, 70 eV) m/z = 159.8 [M + H]^+^, 316.9 [MMH]^+^.

###### 2.5.1.1.2 3,5-Dimethoxybenzylisothiocyanat (2)

3,5-Dimethoxybenzylamin (841 mg, 5.00 mmol) was dissolved in methylene chloride (26 mL) and trimethylamine (2.8 mL, 20.2 mmol) was added. The mixture was stirred at 0°C for 10 min and afterwards, thiophosgene (400 μL, 5.22 mmol) was added dropwise. After stirring for 20 min, the mixture was warmed to room temperature and stirred for further 3 h. The solution was acidified with aqu. HCl (0.1 M) and extracted with methylene chloride. The organic phase was dried over sodium sulfate and the solvent was removed under reduced pressure. The raw product was purified by flash column chromatography on silica gel (5% EA/PE). The product was obtained as a brown solid. Yield: 868 mg (4.15 mmol; 83%). C_10_H_11_NO_2_S (M_*r*_ 209.26). ^1^H NMR (CDCl_3_): *δ* = 6.45 (d, ^4^ *J* = 2.3 Hz, 2 H, C^2/6^_Ar_ *H*), 6.42 (t, ^4^ *J* = 2.3 Hz, 1 H, C^4^_Ar_ *H*), 4.65 (s, 2 H, C*H*_2_), 3.80 (s, 6 H, C^3/5^-OC*H*_3_) ppm. ^13^C-NMR (CDCl_3_): *δ* = 161.2 (*C* ^3/5^_Ar_), 136.5 (*C* ^1^_Ar_), 104.8 (*C* ^2/6^_Ar_H), 100.1 (*C* ^4^_Ar_H), 55.4 (C^3/5^-O*C*H_3_), 48.7 (*C*H_2_) ppm. MS (ESI, 70 eV) m/z = 419.1 [2M + H]^+^.

###### 2.5.1.1.3 3-(3,5-Dimethoxybenzyl)-2-thioxo-2,3,6,7-tetrahydrothieno[3,2-*d*]pyrimidin-4 (1*H*)-on (4)

The compound was obtained from reacting **1** (227 mg, 1.43 mmol) and **2** (327 mg, 1.56 mmol) in pyridine (6 mL). The mixture was heated to 120°C under reflux for 24 h. The solvent was removed under reduced pressure and the raw product purified by flash chromatography on silica gel (gradient EA/PE). The product was obtained as a brown solid. Yield: 173 mg (0.51 mmol; 36%). C_15_H_16_N_2_O_3_S_2_ (M_*r*_ 336.42). ^1^H-NMR (DMSO-*d*_6_): *δ* = 13.23 (s, 1 H, N*H*), 6.41-6.38 (m, 3 H, C^2/6^_Benz_ *H*, C^4^_Benz_ *H*), 5.46 (s, 2 H, N-C*H*_2_), 3.70 (s, 6 H, C^3/5^-OC*H*_3_), 3.38-3.33 (m, 2 H, SCH_2_C*H*_2_), 3.25-3.19 (m, 2 H, SC*H*_2_CH_2_) ppm. ^13^C-NMR (DMSO-d_6_): *δ* = 174.6 (*C* ^2^_Pyrn_), 160.3 (*C* ^3/5^_Benz_), 156.5 (*C* ^4^_Pyrn_), 149.4 (*C* ^6^_Pyrn_), 138.4 (*C* ^1^_Benz_), 114.0 (*C* ^5^_Pyrn_), 105.4 (*C* ^2/6^_Benz_H), 98.2 (*C* ^4^_Benz_H), 55.1 (C^3/5^-O*C*H_3_), 48.8 (N-*C*H_2_), 34.5 (S*C*H_2_CH_2_), 28.9 (SCH_2_ *C*H_2_) ppm. MS (ESI, 70 eV) m/z = 337.0 [M + H]^+^.

###### 2.5.1.1.4 3-Isopropyl-2-thioxo-2,3,6,7-tetrahydrothieno[3,2-*d*]pyrimidin-4(1*H*)-on (5)

The product was obtained starting from **1** (242 mg, 2.51 mmol), isopropyl thiocyanate **3** (190 μL, 1.78 mmol) and potassium tert.-butanolate (282 mg, 2.51 mmol) in DMF (12.5 mL). The mixture was heated to 120°C under reflux for 24 h. It was neutralized with 1 M HCl and the precipitated product was recrystallized from EtOH. The product was obtained as a brown solid. Yield: 144 mg (0.63 mmol; 41%). C_9_H_12_N_2_OS_2_ (M_*r*_ 228.33). ^1^H-NMR (DMSO-*d*_6_): *δ* = 13.02 (s, 1 H, N*H*), 5.94 (qt., ^3^ *J* = 6.9 Hz, 1 H, C*H*(CH_3_)_2_), 3.33-3.28 (m, 2 H, SCH_2_C*H*_2_), 3.18-3.12 (m, 2 H, SC*H*_2_CH_2_), 1.16 (d, ^3^ *J* = 6.9 Hz, 6 H, CH(C*H*_3_)_2_) ppm. ^13^C-NMR (DMSO-d_6_): *δ* = 174.8 (*C* ^2^_Pyrn_), 157.0 (*C* ^4^_Pyrn_), 148.2 (*C* ^6^_Pyrn_), 115.3 (*C* ^5^_Pyrn_), 53.0 (*C*H(CH_3_)_2_), 34.3 (S*C*H_2_CH_2_), 28.7 (SCH_2_ *C*H_2_), 18.3 (CH(*C*H_3_)_2_) ppm. MS (ESI, 70 eV) m/z = 455.2 [2M-H_2_ + H]^+^.

###### 2.5.1.1.5 2-Chloro-N-(6-(trifluormethyl)benzo[*d*]thiazol-2-yl)acetamid (6)

2-Amino-6-(trifluormethyl) benzothiazole (1.19 g, 5.47 mmol) and trimethylamine (0.9 mL, 6.49 mmol) are dissolved in methylene chloride (9 mL). While stirring at room temperature, a solution of 2-chloracetylchloride (0.45 mL, 5.65 mmol) in methylene chloride (3 mL) was added dropwise. Stirring for 24 h, the solvent was removed under reduced pressure and the raw product was recrystallized from EtOH/H_2_O. The product was obtained as colorless needles. Yield: 1.49 g (4.92 mmol; 90%). C_10_H_6_ClF_3_N_2_OS (M_*r*_ 294.68). ^1^H-NMR (DMSO-*d*_6_): *δ* = 12.96 (s, 1 H, N*H*), 8.52 (d, *J*_F H_ = 1.6 Hz, 1 H, C^7^_Bnth_ *H*), 7.94 (d, ^3^ *J* = 8.5 Hz, 1 H, C^4^_Bnth_ *H*), 7.75 (d, ^3^ *J* = 8.5 Hz, 1 H, C^5^_Bnth_ *H*), 4.50 (s, 2 H, C*H*_2_) ppm. ^13^C-NMR (DMSO-d_6_): *δ* = 166.4 (*C*=O), 160.8 (*C* ^2^_Bnth_), 151.2 (*C* ^3a^_Bnth_), 132.0 (*C* ^7a^_Bnth_), 124.5 (d, ^1^ *J*_CF_ = 271.8 Hz, *C*F_3_), 123.9 (d, ^2^ *J*_CF_ = 31.9 Hz, *C* ^6^_Bnth_), 123.0 (d, ^3^ *J*_CF_ = 3.6 Hz, *C* ^5^_Bnth_H), 121.2 (*C* ^4^_Bnth_H), 120.0 (d, ^3^ *J*_CF_ = 4.0 Hz, *C* ^7^_Bnth_H), 42.5 (*C*H_2_) ppm. MS (ESI, 70 eV) m/z = 294.8 [M + H]^+^.

###### 2.5.1.1.6 2-((3-(3,5-Dimethoxybenzyl)-4-oxo-3,4,6,7-tetrahydrothieno[3,2-*d*]pyrimidin-2-yl)thio)-N-(6-(trifluormethyl)benzo [*d*]thiazol-2-yl)acetamid (G1-4)

The synthesis was carried out under inert gas atmosphere. To a solution of **6** (100 mg, 0.34 mmol) and **4** (104 mg, 0.31 mmol) in DMF (3 mL), trimethylamine (130 μL, 0.94 mmol) was added. After stirring at 80°C for 2 h, the reaction was quenched with water and extracted with ethyl acetate. The raw product was purified by flash chromatography and recrystallized from EtOH afterwards. The product was obtained as colorless solid. Yield: 87.4 mg (0.15 mmol; 48%). C_25_H_21_F_3_N_4_O_4_S_3_ (M_*r*_ 594.64). ^1^H-NMR (DMSO-*d*_6_): *δ* = 12.91 (s, 1 H, N*H*), 8.49 (bs, 1 H, C^7^_Bnth_ *H*), 7.92 (d, ^3^ *J* = 8.5 Hz, 1 H, C^4^_Bnth_ *H*), 7.74 (dd, ^3^ *J* = 8.5 Hz, ^4^ *J* = 1.5 Hz, 1 H, C^5^_Bnth_ *H*), 6.45 (t, ^4^ *J* = 2.2 Hz, 1 H, C^4^_Benz_ *H*), 6.39 (t, ^4^ *J* = 2.1 Hz, 2 H, C^2/6^_Benz_ *H*), 5.19 (s, 2 H, N-C*H*_2_), 4.28 (s, 2 H, C*H*_2_C = O), 3.74 (s, 6 H, C^3/5^-OC*H*_3_), 3.27 (t, ^3^ *J* = 8.5 Hz, 2 H, SCH_2_C*H*_2_), 3.04 (t, ^3^ *J* = 8.5 Hz, 2 H, SC*H*_2_CH_2_) ppm. ^13^C-NMR (DMSO-d_6_): *δ* = 167.5 (CH_2_ *C* = O), 161.0 (*C* ^2^_Bnth_), 160.6 (*C* ^3^_Benz_), 160.4 (*C* ^6^_Pyrn_), 158.0 (*C* ^2^_Pyrn_), 157.0 (*C* ^4^_Pyrn_), 151.3 (*C* ^3a^_Bnth_), 137.1 (*C* ^1^_Benz_), 132.0 (*C* ^7a^_Bnth_), 124.5 (d, ^1^ *J*_CF_ = 271.7 Hz, *C*F_3_), 123.8 (d, ^2^ *J*_CF_ = 31.8 Hz, *C* ^6^_Bnth_), 122.9 (d, ^3^ *J*_CF_ = 4.9 Hz, *C* ^5^_Bnth_H), 121.0 (*C* ^4^_Bnth_H), 119.9 (d, ^3^ *J*_CF_ = 4.6 Hz, *C* ^7^_Bnth_H), 119.3 (*C* ^5^_Pyrn_), 105.1 (*C* ^2/6^_Benz_H), 98.8 (*C* ^4^_Benz_H), 55.2 (C^3/5^-O*C*H_3_), 47.0 (N-*C*H_2_), 36.9 (S*C*H_2_CH_2_), 35.9 (*C*H_2_C = O), 28.4 (SCH_2_ *C*H_2_) ppm. MS (ESI, 70 eV) m/z = 595.0 [M + H]^+^. IR: $\tilde{\nu }$ = 3,277, 2,986, 2,837, 1,684, 1,599, 1,545, 1,477, 1,360, 1,321, 1,283, 1,244, 1,206, 1,153, 1,099, 1,082, 843, 764 cm^−1^.

###### 2.5.1.1.7 2-((3-Isopropyl-4-oxo-3,4,6,7-tetrahydrothieno[3,2-*d*]pyrimidin-2-yl)thio)-N-(6-(trifluormethyl)benzo [*d*]thiazol-2-yl)acetamid (G1-5)

The product was obtained from **6** (83.0 mg, 0.28 mmol) and **5** (61.0 mg, 0.27 mmol) with TEA in DMF as a colorless solid. Yield: 77.4 mg (0.16 mmol; 60%). C_19_H_17_F_3_N_4_O_2_S_3_ (M_*r*_ 486.55). ^1^H-NMR (DMSO-*d*_6_): *δ* = 12.91 (s, 1 H, N*H*), 8.48 (dd, ^4^ *J* = 1.3 Hz, *J*_F H_ = 0.6 Hz, 1 H, C^7^_Bnth_ *H*), 7.93 (d, ^3^ *J* = 8.5 Hz, 1 H, C^4^_Bnth_ *H*), 7.74 (dd, ^3^ *J* = 8.5 Hz, ^4^ *J* = 1.5 Hz, 1 H, C^5^_Bnth_ *H*), 4.56 (bs, 1 H, C*H*(CH_3_)_3_), 4.26 (s, 2 H, C*H*_2_C = O), 3.20 (t, ^3^ *J* = 8.6 Hz, 2 H, SCH*2*C*H*_3_), 2.94 (t, ^3^ *J* = 8.6 Hz, 2 H, SCH_2_C*H*_2_), 1.54 (d, ^3^ *J* = 6.7 Hz, 6 H, CH(C*H*_3_)_3_) ppm. ^13^C-NMR (DMSO-d_6_): *δ* = 167.7 (CH_2_ *C* = O), 161.1 (*C* ^2^_Bnth_), 159.2 (*C* ^6^_Pyrn_), 157.4 (*C* ^2^_Pyrn_), 157.2 (*C* ^4^_Pyrn_), 151.3 (*C* ^3a^_Bnth_), 132.0 (*C* ^7a^_Bnth_), 124.5 (d, ^1^ *J*_CF_ = 272.3 Hz, *C*F_3_), 123.7 (d, ^2^ *J*_CF_ = 31.8 Hz, *C* ^6^_Bnth_), 122.9 (d, ^3^ *J*_CF_ = 3.8 Hz, *C* ^5^_Bnth_H), 121.0 (*C* ^4^_Bnth_H), 121.0 (*C* ^5^_Pyrn_), 119.9 (d, ^3^ *J*_CF_ = 3.8 Hz, *C* ^7^_Bnth_H), 53.8 (*C*H(CH_3_)_2_), 36.5 (S*C*H_2_CH_2_), 36.2 (*C*H_2_C = O), 28.2 (SCH_2_ *C*H_2_), 18.8 (CH(*C*H_3_)_2_) ppm. MS (ESI, 70 eV) m/z = 487.0 [M + H]^+^. IR: $\tilde{\nu }$ = 2,976, 2,941, 1,697, 1,680, 1,570, 1,537, 1,487, 1,449, 1,414, 1,317, 1,275, 1,246, 1,161, 1,115, 1,082, 1,051, 982, 883, 826, 764, 719, 671, 646 cm^−1^.

##### 2.5.1.2 Synthesis of G2-4 – G2-6

**FIGURE 2:**
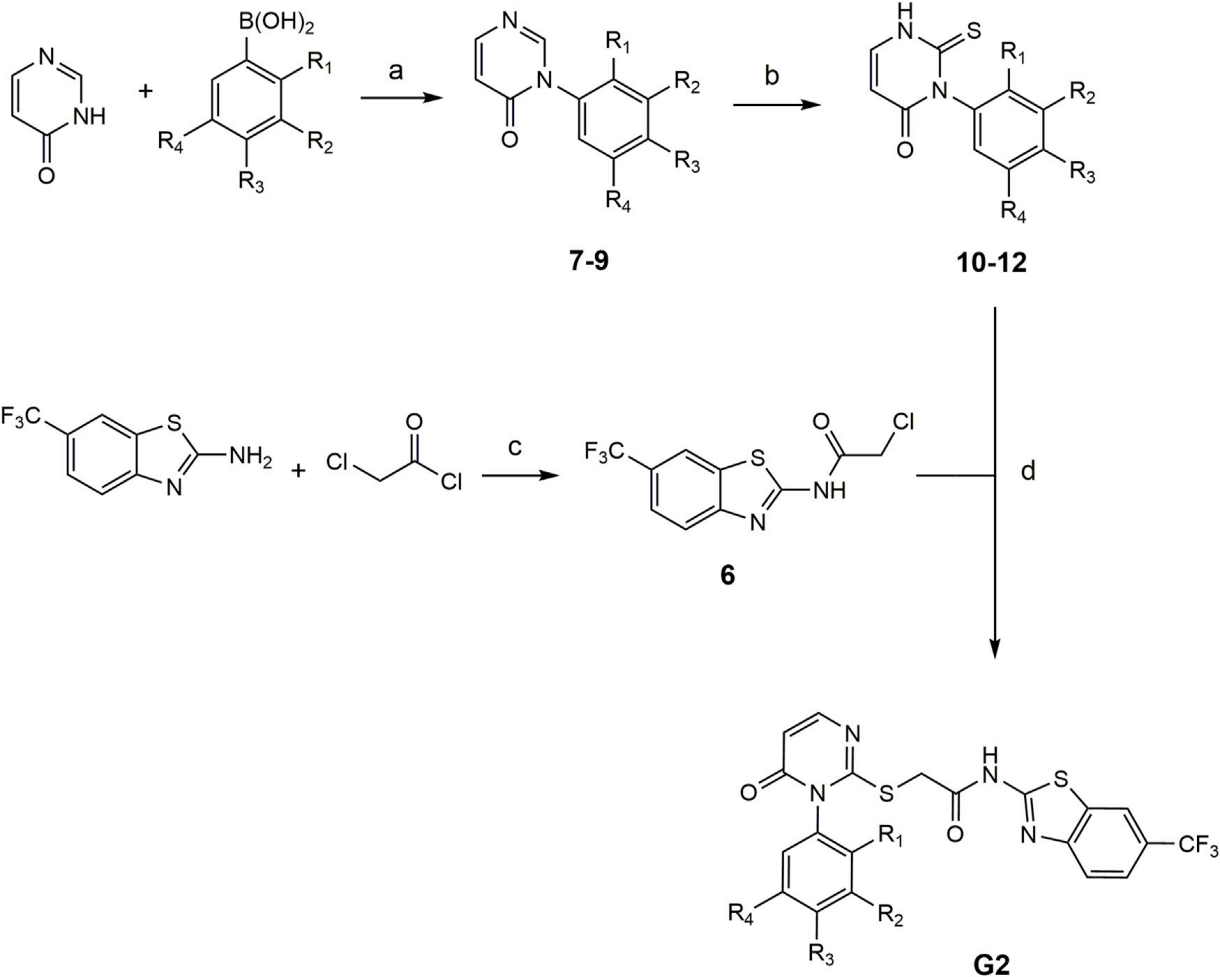
Conditions for the synthesis of compounds G2-4 – G2-6. (a) Pyridine, CuBr, mol sieve (4 Å), DMSO, 90°C, compressed air (b) 1. NaHCO_3_, Et_2_O/H_2_O, rt, 16 h and 2. MeOH, TEA, rt, 16 h (c) inert atm., DMF, TEA, 80°C, 2 h, EA, H_2_O (d) TEA, DMF, 80°C, 2 h: **7**, **10**, **G2-4**: R1 = OCH_3_, R2-R4 = H. **8**, **11**, **G2-5**: R3 = OCH_3_, R1-R2-R4 = H). **9**, **12**, **G2-6** (R2 = OCH_3_, R1-R3-R4 = H).

The compounds G2-4 – G2-6 belong to a class of IWP derivatives previously described by Liu et al. Synthesis of compounds 7-9 was performed based on Chan-Lam coupling from literature which was modified by Liu et al. (2019). In the next reaction step the thioxo group was introduced to phenyl thionochloroformate foloowing the general procedure. The benzothiazole-linker was synthesized after Garcia-Reyes et al. with 2-chloracetylchloride (García-Reyes et al., 2018). The final coupling of the benzothiazole-linker and the pyrimidinone-linker followed previously described IWP derivates (see Figure 2).

###### 2.5.1.2.1 General procedure for the synthesis of 7-9

Molecular sieve (4 Å) was dried *in vacuo*. A mixture of 4-(3H)-pyrimidinone, the appropriate methoxyphenylboronic acid, copper(I)-bromide and the dried molecular sieves 4 Å was dissolved in DMSO. Pyridine was added and the reaction mixture was heated to 90°C under reflux for 4 h. While stirring, compressed air was introduced via a small tube. After stirring at 90°C for 4 h, the mixture was cooled and filtered. After adding water to the filtrate, the solution was extracted with ethyl acetate (EA). The organic phase was washed with water and brine, dried over sodium sulfate and the solvent was removed under reduced pressure. The raw product was purified by column flash-chromatography on silica gel (gradient EA/PE). Synthesis has been described by Liu et al. (2019).

###### 2.5.1.2.2 3-(2-Methoxyphenyl)pyrimidin-4(3H)-one (7)

3-(2-Methoxyphenyl)pyrimidin-4(3H)-one (**7**) was obtained from 4(3*H*)-pyrimidinone (193 mg, 2.01 mmol), 2-methoxyphenylboronic acid (609 mg, 4.01 mmol), copper(I)-bromide (77.3 mg, 0.54 mmol) and pyridine (330 μL, 4.05 mmol) in DMSO (32 mL) as colorless solid. Yield: 144 mg (0.71 mmol; 35%). C_11_H_10_N_2_O_2_ (M_*r*_ 202.21). ^1^H-NMR (DMSO-*d*_6_): *δ* = 8.31 (s, 1 H, C^2^_Pyr_ *H*), 7.97 (d, ^3^ *J* = 6.7 Hz, 1 H, C^6^_Pyr_ *H*), 7.50 (ddd, ^3^ *J* = 8.3 Hz, ^3^ *J* = 7.5 Hz, ^4^ *J* = 1.6 Hz, 1 H, C^4^_Phen_ *H*), 7.38 (dd, ^3^ *J* = 7.7 Hz, ^4^ *J* = 1.6 Hz, 1 H, C^6^_Phen_ *H*), 7.24 (dd, ^3^ *J* = 8.4 Hz, ^4^ *J* = 0.9 Hz, 1 H, C^3^_Phen_ *H*), 6.49 (dd, ^3^ *J* = 6.7 Hz, ^5^ *J* = 0.9 Hz, 1 H, C^5^_Pyr_ *H*), 3.77 (s, 3 H, OC*H*_3_) ppm. ^13^C-NMR (DMSO-d_6_): *δ* = 159.6 (*C* ^4^_Pyrn_), 154.2 (*C* ^2^_Phen_), 153.6 (*C* ^6^_Pyrn_H), 153.0 (*C* ^2^_Pyrn_H), 130.9 (*C* ^4^_Phen_H), 129.0 (*C* ^6^_Phen_H), 125.6 (*C* ^1^_Phen_), 120.6 (*C* ^5^_Phen_H), 115.7 (*C* ^5^_Pyrn_H), 112.6 (*C* ^3^_Phen_H), 55.9 (O*C*H_3_) ppm. MS (ESI, 70 eV) m/z = 203.1 [M + H]^+^.

###### 2.5.1.2.3 3-(4-Methoxyphenyl)pyrimidin-4(3H)-one (8)

3-(4-Methoxyphenyl)pyrimidin-4(3H)-one (**8**) was obtained from 4(3*H*)-pyrimidinone (201 mg, 2.09 mmol), 4-methoxyphenylboronic acid (614 mg, 4.04 mmol), copper(I)-bromide (83.9 mg, 0.58 mmol) and pyridine (330 μL, 4.05 mmol) in DMSO (32 mL) as colorless solid. Yield: 308 mg (1.52 mmol; 73%). C_11_H_10_N_2_O_2_ (M_*r*_ 202.21). ^1^H-NMR (DMSO-*d*_6_): *δ* = 8.40 (s, 1 H, C^2^_Pyr_ *H*), 7.97 (d, ^3^ *J* = 6.7 Hz, 1 H, C^6^_Pyr_ *H*), 7.38 (d, ^3^ *J* = 8.9 Hz, 2 H, C^2/6^_Phen_ *H*), 7.07 (d, ^3^ *J* = 8.9 Hz, 2 H, C^3/5^_Phen_ *H*), 6.49 (dd, ^3^ *J* = 6.7 Hz, ^5^ *J* = 0.8 Hz, 1 H, C^5^_Pyr_ *H*), 3.81 (s, 3 H, OC*H*_3_) ppm. ^13^C-NMR (DMSO-d_6_): *δ* = 160.1 (*C* ^4^_Pyrn_), 159.4 (*C* ^4^_Phen_), 153.5 (*C* ^6^_Pyrn_H), 152.3 (*C* ^2^_Pyrn_H), 129.8 (*C* ^1^_Phen_), 128.3 (*C* ^2/6^_Phen_H), 115.5 (*C* ^5^_Pyrn_H), 114.3 (*C* ^3/5^_Phen_H), 55.5 (O*C*H_3_) ppm. MS (ESI, 70 eV) m/z = 203.1 [M + H]^+^.

###### 2.5.1.2.4 3-(3-Methoxyphenyl)pyrimidin-4(3H)-one (9)

3-(3-Methoxyphenyl)pyrimidin-4(3H)-one (**9**) was obtained from 4(3*H*)-pyrimidinone (193 mg, 2.01 mmol), 3-methoxyphenylboronic acid (610 mg, 4.01 mmol), copper(I)-bromide (61.0 mg, 0.43 mmol) and pyridine (330 μL, 4.05 mmol) in DMSO (32 mL) as colorless solid. Yield: 267 mg (1.32 mmol; 66%). C_11_H_10_N_2_O_2_ (M_*r*_ 202.21). ^1^H-NMR (DMSO-*d*_6_): *δ* = 8.40 (s, 1 H, C^2^_Pyr_ *H*), 7.97 (d, ^3^ *J* = 6.7 Hz, 1 H, C^6^_Pyr_ *H*), 7.38 (d, ^3^ *J* = 8.9 Hz, 2 H, C^2/6^_Phen_ *H*), 7.07 (d, ^3^ *J* = 8.9 Hz, 2 H, C^3/5^_Phen_ *H*), 6.49 (dd, ^3^ *J* = 6.7 Hz, ^5^ *J* = 0.8 Hz, 1 H, C^5^_Pyr_ *H*), 3.81 (s, 3 H, OC*H*_3_) ppm. ^13^C-NMR (DMSO-d_6_): *δ* = 160.1 (*C* ^4^_Pyrn_), 159.4 (*C* ^4^_Phen_), 153.5 (*C* ^6^_Pyrn_H), 152.3 (*C* ^2^_Pyrn_H), 129.8 (*C* ^1^_Phen_), 128.3 (*C* ^2/6^_Phen_H), 115.5 (*C* ^5^_Pyrn_H), 114.3 (*C* ^3/5^_Phen_H), 55.5 (O*C*H_3_) ppm. MS (ESI, 70 eV) m/z = 203.1 [M + H]^+^.

##### 2.5.1.3 General procedure for the synthesis of compounds 10-12

For the first reaction step, respective compounds **7** - **9** and sodium bicarbonate were dissolved in a mixture of 1:1 diethyl ether/EA and water. After adding phenyl thionochloroformate, the mixture was stirred for 16 h at room temperature. It was then extracted with EA and the organic phase was washed with brine, dried over sodium sulfate and the solvent was removed under reduced pressure. In the second reaction step the raw product was dissolved in methanol and trimethylamine was added. The mixture was stirred for 16 h at room temperature before the solvent was removed under pressure. The raw product was purified by flash chromatography on silica gel.

###### 2.5.1.3.1 3-(2-Methoxyphenyl)-2-thioxo-2,3-dihydropyrimidin-4(1*H*)-one (10)

3-(2-Methoxyphenyl)-2-thioxo-2,3-dihydropyrimidin-4(1*H*)-one (**10**) was obtained from **7** (118 mg, 0.59 mmol), sodium bicarbonate (307 mg, 3.65 mmol), phenyl thionochloroformate (210 μL, 1.51 mmol) in a mixture of 1:1 diethyl ether/EA (10 mL), water (5 mL), methanol (10 mL) and triethylamine (258 μL, 1.86 mmol). It was purified by flash chromatography on silica gel (50% EA/PE) as a pale-yellow solid. Yield: 101 mg (0.43 mmol; 74%). C_11_H_10_N_2_O_2_S (M_*r*_ 234.27). ^1^H-NMR (DMSO-d_6_): *δ* = 12.62 (mc, 1 H, N-*H*), 7.52 (dd, ^3^ *J* = 7.6 Hz, ^3^ *J* = 5.6 Hz, 1 H, C^6^_Pyrn_ *H*), 7.37 (ddd, ^3^ *J* = 8.2 Hz, ^3^ *J* = 7.5 Hz, ^4^ *J* = 1.5 Hz, 1 H, C^4^_Phen_ *H*), 7.13-7.11 (m, 2 H, C^3^_Phen_ *H*, C^6^_Phen_ *H*), 6.99 (dt, ^3^ *J* = 7.5 Hz, ^4^ *J* = 1.0 Hz, 1 H, C^5^_Phen_ *H*), 6.00 (d, ^3^ *J* = 7.5 Hz, 1 H, C^5^_Pyrn_ *H*), 3.72 (s, 3 H, C^2^-OC*H*_3_) ppm. ^13^C-NMR (DMSO-d_6_): *δ* = 177.6 (*C* ^2^_Pyrn_), 160.2 (*C* ^4^_Pyrn_), 154.1 (*C* ^2^_Phen_), 141.4 (*C* ^6^_Pyrn_ *H*), 129.8 (*C* ^4^_Phen_H), 129.6 (*C* ^6^_Phen_H), 127.5 (*C* ^1^_Phen_), 120.6 (*C* ^5^_Phen_H), 112.3 (*C* ^3^_Phen_H), 104.5 (*C* ^5^_Pyrn_H), 55.7 (C^2^-OC*H*_3_) ppm. MS (ESI, 70 eV) m/z = 235.0 [M + H]^+^.

###### 2.5.1.3.2 3-(4-Methoxyphenyl)-2-thioxo-2,3-dihydropyrimidin-4(1*H*)-one (11)

3-(4-Methoxyphenyl)-2-thioxo-2,3-dihydropyrimidin-4(1*H*)-one (**11**) was obtained from **8** (206 mg, 1.02 mmol), sodium bicarbonate (527 mg, 6.27 mmol), phenyl thionochloroformate (353 μL, 2.54 mmol) in a mixture of 1:1 diethyl ether/EA (8 mL), water (8 mL), methanol (22 mL) and triethylamine (430 μL, 3.10 mmol). It was purified by flash chromatography on silica gel (gradient EA/PE) as a colorless solid. Yield: 170 mg (0.73 mmol; 71%). C_11_H_10_N_2_O_2_S (M_*r*_ 234.27). ^1^H-NMR (DMSO-d_6_): *δ* = 12.61 (s, 1 H, N-*H*), 7.51 (d, ^3^ *J* = 7.6 Hz, 1 H, C^6^_Pyrn_ *H*), 7.08 (d, ^3^ *J* = 8.9 Hz, 2 H, C^2/6^_Phen_ *H*), 6.98 (d, ^3^ *J* = 8.9 Hz, 2 H, C^3/5^_Phen_ *H*), 6.02 (d, ^3^ *J* = 7.6 Hz, 1 H, C^5^_Pyrn_ *H*), 3.79 (s, 3 H, C^2^-OC*H*_3_) ppm. ^13^C-NMR (DMSO-d_6_): *δ* = 178.1 (*C* ^2^_Pyrn_), 160.9 (*C* ^4^_Pyrn_), 158.7 (*C* ^4^_Phen_), 141.2 (*C* ^6^_Pyrn_H), 131.8 (*C* ^1^_Phen_), 129.5 (*C* ^2/6^_Phen_H), 114.3 (*C* ^3/5^_Phen_H), 104.9 (*C* ^5^_Pyrn_H), 55.3 (C^4^-OC*H*_3_) ppm. MS (ESI, 70 eV) m/z = 235.0 [M + H]^+^.

###### 2.5.1.3.3 3-(3-Methoxyphenyl)-2-thioxo-2,3-dihydropyrimidin-4(1*H*)-one (12)

3-(3-Methoxyphenyl)-2-thioxo-2,3-dihydropyrimidin-4(1*H*)-one (**12**) was obtained from **9** (205 mg, 1.01 mmol), sodium bicarbonate (565 mg, 6.73 mmol), phenyl thionochloroformate (353 μL, 2.54 mmol) in a mixture of 1:1 diethyl ether/EA (8 mL), water (8 mL), methanol (20 mL) and triethylamine (430 μL, 3.10 mmol). It was purified by flash chromatography on silica gel (gradient EA/PE) as a yellow solid. Yield: 183 mg (0.78 mmol; 77%). C_11_H_10_N_2_O_2_S (M_*r*_ 234.27). ^1^H-NMR (DMSO-d_6_): *δ* = 12.65 (s, 1 H, N-*H*), 7.52-7.36 (m, 2 H, C^6^_Pyrn_ *H*, C^2^_Phen_ *H*), 6.96-6.74 (m, 3 H, C^4^_Phen_ *H*, C^5^_Phen_ *H*, C^6^_Phen_ *H*), 6.03 (d, ^3^ *J* = 6.5 Hz, 1 H, C^5^_Pyrn_ *H*), 3.75 (s, 3 H, C^3^-OC*H*_3_) ppm. ^13^C-NMR (DMSO-d_6_): *δ* = 178.1 (*C* ^2^_Pyrn_), 161.1 (*C* ^4^_Pyrn_), 160.4 (*C* ^3^_Phen_), 141.7 (*C* ^6^_Pyrn_H), 140.6 (*C* ^1^_Phen_), 130.2 (*C* ^5^_Phen_H), 121.1 (*C* ^6^_Phen_H), 114.1 (*C* ^2^_Phen_H), 105.4 (*C* ^5^_Pyrn_H), 55.7 (C^3^-OC*H*_3_) ppm. MS (ESI, 70 eV) m/z = 235.0 [M + H]^+^.

##### 2.5.1.4 General procedure for the synthesis of compounds G2-4 – G2-6

The synthesis was carried out under inert gas atmosphere. To a solution of **6** and respective compounds **10–12** in DMF, trimethylamine was added. After stirring at 80°C for 2 h, the reaction was quenched with water and extracted with ethyl acetate. The raw product was purified by flash chromatography on silica gel and if necessary, recrystallized from EtOH/water afterwards.

###### 2.5.1.4.1 2-((3-(2-Methoxyphenyl)-4-oxo-3,4-dihydropyrimidin-2-yl)thio)-*N*-(6-(trifluormethyl)benzo [*d*]thiazol-2-yl)acetamid G2-4

2-((3-(2-Methoxyphenyl)-4-oxo-3,4-dihydropyrimidin-2-yl)thio)-*N*-(6-(trifluormethyl)benzo[*d*]thiazol-2-yl)acetamid was obtained from reacting **6** (112 mg, 0.38 mmol), **10** (85.6 mg, 0.37 mmol) and trimethylamine (150 μL, 1.08 mmol) in DMF (4 mL). The raw product was purified by flash chromatography on silica gel (40% EA/PE) as a pale-yellow solid. Yield: 170 mg (0.35 mmol; 95%). C_21_H_15_F_3_N_4_O_3_S_2_ (M_*r*_ 492.49). ^1^H-NMR (DMSO-*d*_6_): *δ* = 12.88 (s, 1 H, N*H*), 8.50 (bs, 1 H, C^7^_Bnth_ *H*), 7.92 (d, ^3^ *J* = 8.5 Hz, 1 H, C^4^_Bnth_ *H*), 7.84 (d, ^3^ *J* = 6.6 Hz, 1 H, C^6^_Pyrn_ *H*), 7.74 (dd, ^3^ *J* = 8.6 Hz, ^4^ *J* = 1.6 Hz, 1 H, C^5^_Bnth_ *H*), 7.56 (mc, 1 H, C^4^_Phen_ *H*), 7.37 (dd, ^3^ *J* = 7.7 Hz, ^4^ *J* = 1.7 Hz, 1 H, C^6^_Phen_ *H*), 7.28 (dd, ^3^ *J* = 8.4 Hz, ^4^ *J* = 0.9 Hz, 1 H, C^3^_Phen_ *H*), 7.13 (dt, ^3^ *J* = 7.6 Hz, ^4^ *J* = 1.1 Hz, 1 H, C^5^_Phen_ *H*), 6.26 (d, ^3^ *J* = 6.6 Hz, 1 H, C^5^_Pyrn_ *H*), 4.20 (d, ^4^ *J* = 1.4 Hz, 2 H, C*H*_2_C = O), 3.80 (s, 3 H, C^2^_Phen_-OC*H*_3_) ppm. ^13^C-NMR (DMSO-d_6_): *δ* = 167.4 (CH_2_ *C* = O), 163.1 (*C* ^2^_Pyrn_), 161.0 (*C* ^2^_Bnth_), 160.2 (*C* ^4^_Pyrn_), 154.6 (*C* ^2^_Phen_), 152.5 (*C* ^6^_Pyrn_H), 151.3 (*C* ^3a^_Bnth_), 132.1 (*C* ^4^_Phen_H), 132.0 (*C* ^7a^_Bnth_), 124.6 (d, ^1^ *J*_CF_ = 271.9 Hz, *C*F_3_), 123.8 (d, ^2^ *J*_CF_ = 31.4 Hz, *C* ^6^_Bnth_), 123.7 (*C* ^1^_Phen_), 123.0 (d, ^3^ *J*_CF_ = 3.9 Hz, *C* ^5^_Bnth_H), 121.1 (*C* ^5^_Phen_H), 121.06 (*C* ^4^_Bnth_H), 120.0 (d, ^3^ *J*_CF_ = 4.9 Hz, *C* ^7^_Bnth_H), 113.0 (*C* ^3^_Phen_H), 110.8 (*C* ^5^_Pyrn_H), 56.0 (C^2^_Phen_-O*C*H_3_), 35.8 (*C*H_2_C = O) ppm. MS (ESI, 70 eV) m/z = 492.9 [M + H]^+^. IR: $\tilde{\nu }$ = 2,957, 2,924, 2,855, 1,668, 1,603, 1,541, 1,485, 1,414, 1,317, 1,261, 1,161, 1,117, 1,082, 1,022, 995, 883, 824, 748, 718, 677, 646 cm^−1^.

###### 2.5.1.4.2 2-((3-(4-Methoxyphenyl)-4-oxo-3,4-dihydropyrimidin-2-yl)thio)-*N*-(6-(trifluormethyl)benzo[*d*]thiazol-2-yl)acetamid G2-5

2-((3-(4-Methoxyphenyl)-4-oxo-3,4-dihydropyrimidin-2-yl)thio)-*N*-(6-(trifluormethyl)benzo[*d*]thiazol-2-yl)acetamid was obtained from reacting **6** (161 mg, 0.55 mmol), **11** (123 mg, 0.53 mmol) and trimethylamine (217 μL, 1.57 mmol) in DMF (6 mL). The raw product was purified by flash chromatography on silica gel (gradient EA/PE) and recrystallized from EtOH/water as a colorless solid. Yield: 183 mg (0.37 mmol; 71%). C_21_H_15_F_3_N_4_O_3_S_2_ (M_*r*_ 492.49). ^1^H-NMR (DMSO-*d*_6_): *δ* = 12.89 (s, 1 H, N*H*), 8.50 (bs, 1 H, C^7^_Bnth_ *H*), 7.92 (d, ^3^ *J* = 8.5 Hz, 1 H, C^4^_Bnth_ *H*), 7.83 (d, ^3^ *J* = 6.6 Hz, 1 H, C^6^_Pyrn_ *H*), 7.75 (dd, ^3^ *J* = 8.9 Hz, ^4^ *J* = 1.7 Hz, 1 H, C^5^_Bnth_ *H*), 7.34 (d, ^3^ *J* = 9.0 Hz, 2 H, C^2/6^_Phen_ *H*), 7.12 (d, ^3^ *J* = 8.9 Hz, 2 H, C^3/5^_Phen_ *H*), 6.26 (d, ^3^ *J* = 6.6 Hz, 1 H, C^5^_Pyrn_ *H*), 4.20 (s, 2 H, C*H*_2_C = O), 3.84 (s, 3 H, C^4^_Phen_-OC*H*_3_) ppm. ^13^C-NMR (DMSO-d_6_): *δ* = 167.5 (CH_2_ *C* = O), 163.2 (*C* ^2^_Pyrn_), 161.0 (*C* ^2^_Bnth_), 160.9 (*C* ^4^_Pyrn_), 160.3 (*C* ^4^_Phen_), 152.3 (*C* ^6^_Pyrn_H), 151.3 (*C* ^3a^_Bnth_), 132.0 (*C* ^7a^_Bnth_), 130.0 (*C* ^2/6^_Phen_H), 128.0 (*C* ^1^_Phen_), 124.6 (d, ^1^ *J*_CF_ = 272.3 Hz, *C*F_3_), 123.8 (d, ^2^ *J*_CF_ = 31.9 Hz, *C* ^6^_Bnth_), 123.1 (d, ^3^ *J*_CF_ = 4.2 Hz, *C* ^5^_Bnth_H), 121.1 (*C* ^4^_Bnth_H), 120.0 (d, ^3^ *J*_CF_ = 4.1 Hz, *C* ^7^_Bnth_H), 114.9 (*C* ^3/5^_Phen_H), 110.9 (*C* ^5^_Pyrn_H), 55.5 (C^4^_Phen_-O*C*H_3_), 36.1 (*C*H_2_C = O) ppm. MS (ESI, 70 eV) m/z = 492.9 [M + H]^+^. IR: $\tilde{\nu }$ = 3,071, 2,970, 2,841, 1,699, 1,605, 1,591, 1,568, 1,541, 1,510, 1,487, 1,464, 1,414, 1,398, 1,337, 1,321, 1,302, 1,275, 1,248, 1,180, 1,173, 1,157, 1,136, 1,115, 1,082, 1,051, 1,024, 1,015, 999, 905, 849, 831, 719, 665 cm^−1^.

###### 2.5.1.4.3 2-((3-(3-Methoxyphenyl)-4-oxo-3,4-dihydropyrimidin-2-yl)thio)-*N*-(6-(trifluormethyl)benzo[*d*]thiazol-2-yl)acetamid (G2-6)

2-((3-(3-Methoxyphenyl)-4-oxo-3,4-dihydropyrimidin-2-yl)thio)-*N*-(6-(trifluormethyl)benzo[*d*]thiazol-2-yl)acetamid was obtained from reacting **6** (171 mg, 0.58 mmol), **12** (130 mg, 0.55 mmol) and trimethylamine (229 μL, 1.65 mmol) in DMF (6 mL). The raw product was purified by flash chromatography on silica gel (gradient EA/PE) and recrystallized from EtOH/water as a pale-yellow solid. Yield: 230 mg (0.47 mmol; 84%). C_21_H_15_F_3_N_4_O_3_S_2_ (M_*r*_ 492.49). ^1^H-NMR (DMSO-*d*_6_): *δ* = 12.89 (s, 1 H, N*H*), 8.50 (bs, 1 H, C^7^_Bnth_ *H*), 7.92 (d, ^3^ *J* = 8.5 Hz, 1 H, C^4^_Bnth_ *H*), 7.84 (d, ^3^ *J* = 6.6 Hz, 1 H, C^6^_Pyrn_ *H*), 7.75 (dd, ^3^ *J* = 8.6 Hz, ^4^ *J* = 1.6 Hz, 1 H, C^5^_Bnth_ *H*), 7.51 (t, ^3^ *J* = 8.1 Hz, 1 H, C^5^_Phen_ *H*), 7.15 (m_c_, 1 H, C^6^_Phen_ *H*), 7.07 (t, ^4^ *J* = 2.1 Hz, 1 H, C^2^_Phen_ *H*), 7.00 (m_c_, 1 H, C^4^_Phen_ *H*), 6.28 (d, ^3^ *J* = 6.6 Hz, 1 H, C^5^_Pyrn_ *H*), 4.21 (s, 2 H, C*H*_2_C = O), 3.81 (s, 3 H, C^3^_Phen_-OC*H*_3_) ppm. ^13^C-NMR (DMSO-d_6_): *δ* = 167.5 (CH_2_ *C* = O), 162.6 (*C* ^2^_Pyrn_), 161.1 (*C* ^2^_Bnth_), 160.6 (*C* ^4^_Pyrn_), 160.2 (*C* ^3^_Phen_), 152.3 (*C* ^6^_Pyrn_H), 151.3 (*C* ^3a^_Bnth_), 136.6 (*C* ^1^_Phen_), 132.0 (*C* ^7a^_Bnth_), 130.6 (*C* ^5^_Phen_H), 124.6 (d, ^1^ *J*_CF_ = 271.4 Hz, *C*F_3_), 123.8 (d, ^2^ *J*_CF_ = 32.4 Hz, *C* ^6^_Bnth_), 123.0 (d, ^3^ *J*_CF_ = 3.3 Hz, *C* ^5^_Bnth_H), 121.1 (*C* ^4^_Bnth_H), 120.7 (*C* ^4^_Phen_H), 120.0 (d, ^3^ *J*_CF_ = 4.3 Hz, *C* ^7^_Bnth_H), 115.8 (*C* ^6^_Phen_H), 114.4 (*C* ^2^_Phen_H), 111.1 (*C* ^5^_Pyrn_H), 55.6 (C^3^_Phen_-O*C*H_3_), 36.0 (*C*H_2_C = O) ppm. MS (ESI, 70 eV) m/z = 492.9 [M + H]^+^. IR: $\tilde{\nu }$ = 3,140, 3,059, 2,967, 2,936, 2,363, 1714, 1,667, 1,607, 1,568, 1,553, 1,483, 1,466, 1,416, 1,369, 1,315, 1,285, 1,265, 1,250, 1,192, 1,159, 1,152, 1,132, 1,113, 1,080, 1,032, 1,011, 980, 961, 835, 829, 789, 718, 691, 677, 646 cm^−1^.

### 2.6 Molecular modelling

Molecular modelling was performed on a DELL Precision T3610 four-core workstation using Schrödinger Maestro, version 12.6.1244 (Schrödinger LLC, New York, NY, United States, 2020-4). A homology model of zebrafish CK1δ was generated based on a ligand-protein structure of human CK1δ [PDB code 5OKT (García-Reyes et al., 2018)] using the default workflow in Maestro Prime. The homology model was further prepared with the Protein Preparation Wizard regarding assignment of bond orders, addition of hydrogen atoms, identification of disulfide bonds and conversion of artificial selenomethionines to methionines (default settings). Designed ligands were minimized with MacroModel, receptor grid generation and Ligand docking (Glide SP) were performed with Glide, using the standard protocol. The interaction of the most potent inhibitors G2-2 and G2-3 with the zebrafish CK1δ variants A and B was compared to the ligand-protein model of G2-2 with human CK1δ.

### 2.7 Zebrafish care and breeding, microinjection and pharmacological treatment

All procedures and experiments in this study were carried out after appropriate institutional approvals (Tierforschungszentrum (TFZ) der Universität Ulm, No. z.183), which conforms to the EU Directive 2010/63/EU. Care and breeding of zebrafish (*D. rerio*) were carried out as previously described (Westerfield, 1993; Keßler et al., 2015). The TüAB wildtype strain was used for all experiments. Morpholino-modified antisense oligonucleotides (MOs; Gene Tools, LLC, Oregon, United States) were injected into the yolk of fertilized zebrafish oocytes. To knockdown *csnk1da* and *csnk1db*, MOs targeting the translational start site were used. 400 µM *csnk1da* morpholino (*csnk1da* MO) (5′-TCG​GTT​TCC​TAC​TCT​CAA​TTC​CAT​G-3′) and 300 µM *csnk1db* morpholino (*csnk1db* MO) (5′-CTG​TTT​CCA​ACT​CGT​AGC​TCC​ATT​G-3′) were injected, standard control morpholino (Std Ctrl MO) (5′- CCT​CTT​ACC​TCA​GTT​ACA​ATT​TAT​A -3′) served as a control. Zebrafish embryos were dechorionated at 24 h post fertilization (hpf) and treated with the respective concentration of inhibitor, dissolved in DMSO (Sigma-Aldrich, Stadt, Land) and diluted in E3 medium (5 mM NaCl, 0.17 mM KCl, 0.33 mM CaCl_2_, 0.33 mM MgSO_4_, 0.6 µM Methylene Blue dissolved in water). DMSO served as solvent control. Embryos were treated from 24 hpf until 72 hpf, the compound solution was renewed daily. Pictures and movies were recorded at 48 and 72 hpf. Embryos were immobilized in 2.5% methylcellulose. Pictures were taken on an Olympus stereo microscope SZX16 and movies were recorded on an inverse Leica stereo microscope DM IL LED Fluo equipped with a FLEXACAM C1.

### 2.8 Statistical methods

Statistical analysis and data evaluation were performed using the software MS Excel (Microsoft Corporation, Stadt, United States) and Prism 8 (GraphPad, San Diego, United States). All experiments were conducted in triplicates and the data is presented as (normalized) mean ± standard deviation.

## 3 Results

### 3.1 CK1δ is highly conserved between human and zebrafish

**FIGURE 3:**
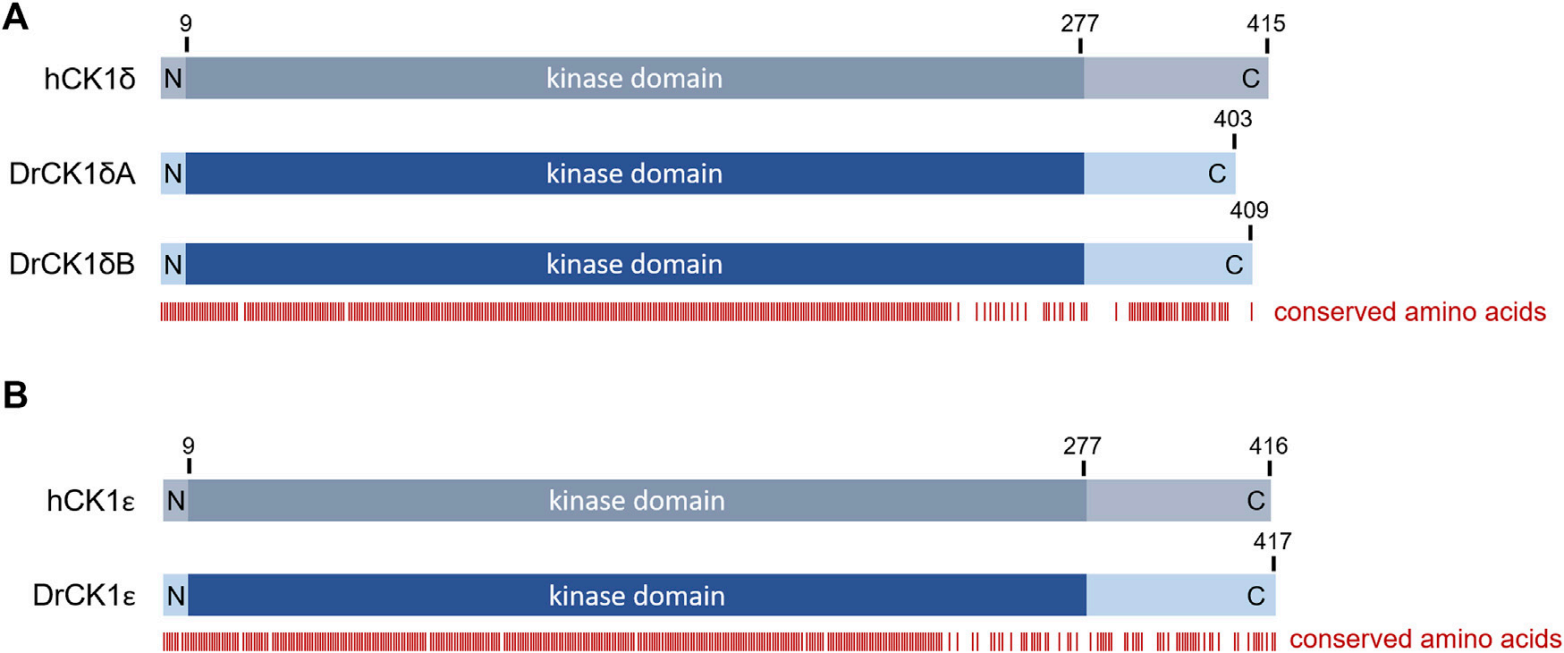
Overview over the amino acid sequence conservation of the human and zebrafish CK1 variants. Alignments between human CK1 and the zebrafish CK1 variants were performed using PRALINE multiple sequence alignment (Centre for Integrative Bioinformatics VU, see also Supplementary Figure S1). Based on the alignment, the figure shows the conservation of certain amino acids indicated by the red bars below. There is a very high conservation visible in the N-terminal and kinase domains whereas the C-terminal region shows more variability **(A)** Alignment between human CK1δ (hCK1δ) and zebrafish CK1δA and B (DrCK1δA and DrCK1δB). **(B)** Alignment between human CK1ε (hCK1ε) and zebrafish CK1ε (DrCK1ε).

The zebrafish (*D. rerio*) is a valuable model to investigate effects of SMIs *in vivo* (Peterson et al., 2000; Keßler et al., 2015), with a conservation of around 70% of protein-coding genes compared to humans (Howe et al., 2013; Bradford et al., 2017). The alignment of human CK1δ with zebrafish CK1δA and B revealed high conservation of the kinase domain (amino acids 9–277) and a 100% identity in the ATP binding side (amino acids 15–24), suggesting similar ATP- and SMI-binding properties. However, the C-terminal domain, involved in regulatory functions, like contribution to binding phosphorylated substrates through its W1 motif or autoinhibition (Graves and Roach, 1995; Longenecker et al., 1996; Harold et al., 2023), presents a high order of variation (Figure 3; Supplementary Figure S1) that could potentially be the source of different enzymatic behavior.

### 3.2 Zebrafish CK1 isoforms are kinetically comparable to their human CK1 counterparts

**FIGURE 4:**
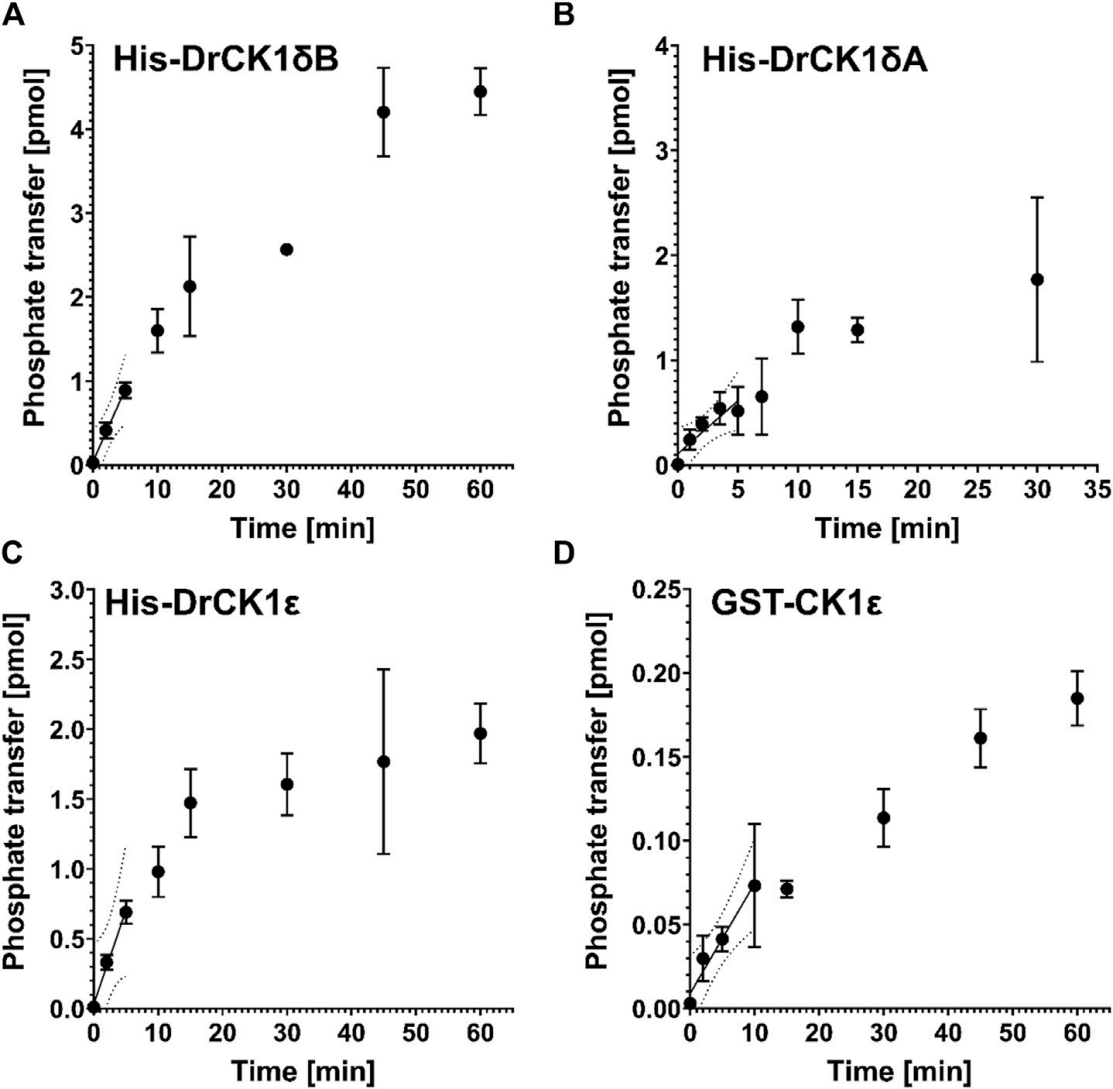
Product-over-time progression curves for α-casein phosphorylation mediated by the zebrafish CK1 isoforms and GST-CK1ε. The time-dependent phosphorylation of α-casein, catalyzed by **(A)** 70 nM His-DrCK1δA, **(B)** 33 nM His-DrCK1δB, **(C)** 7 nM His-DrCK1ε **(D)** and 70 nM GST-CK1ε was determined in *in vitro* kinase assays. The ATP-concentration of the reaction mix was 10 μM and 2 g/L substrate were utilized. The assays were performed in presence of the inhibitor solvent DMSO. Linear regression was performed to determine the maximum coefficient of determination (*R* ^2^) and to identify the initial velocity region (Roth et al., 2021). Product-over-time progression curve for GST-CK1δ was established by Roth et al. (Figures 3, 4) and the data is licensed under a Creative Commons Attribution 4.0 International License (CC BY 4.0) (Roth et al., 2021).

To ensure comparability between the results of kinase assays, Roth et al. suggested a workflow to establish robust standard conditions (Roth et al., 2021). After having determined the optimal kinase concentrations for phosphorylation of α-casein by His-DrCK1δA (70 nM), His-DrCK1δB (33 nM) and His-DrCK1ε (7 nM) in *in vitro* kinase assays (Supplementary Table S3), the performed product-over-time curves at the optimal concentration of the respective kinase show the characteristic linear region in the beginning, where the initial velocity is maximal. At later time points, the slope is decreasing, and phosphate transfer approaches a plateau (Figure 4). To determine the ideal reaction time, a linear regression analysis was performed for each kinase. The results presented in Supplementary Table S4 indicate that the ideal reaction time of His-DrCK1δA, His-DrCK1δB, His-DrCK1ε, and GST-CK1δ is ranging between 5 and 15 min.

**FIGURE 5:**
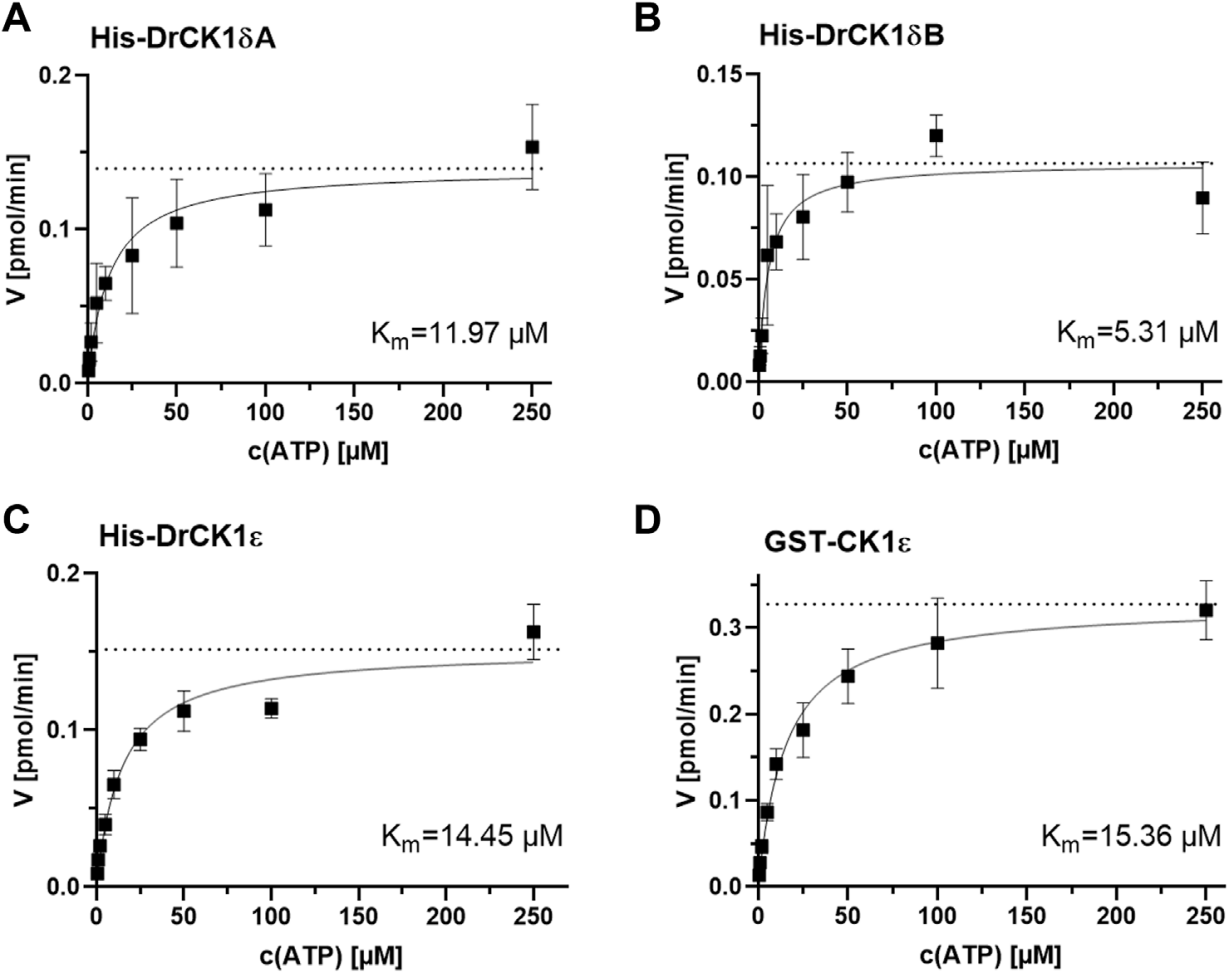
Determination of the kinase-specific K_m_(ATP) by evaluation of the Michaelis-Menten enzyme kinetic. The K_m_(ATP) was assessed by performing *in vitro* kinase assays with different ATP-concentrations (0.5, 1, 2, 5, 10, 25, 50, 100 and 250 µM) and by analyzing the resulting Michaelis-Menten kinetics. The K_m_(ATP) describes the concentration of the phosphate-donor ATP at which half of the maximal reaction velocity (V_max_, marked by the dashed line) is reached. **(A)** Michaelis-Menten kinetic for His-DrCK1δA, K_m_(ATP) = 11.97 µM. **(B)** Michaelis-Menten kinetic for His-DrCK1δB, K_m_(ATP) = 5.31 µM. **(C)** Michaelis-Menten kinetic for His-DrCK1ε, K_m_(ATP) = 14.45 µM. **(D)** Michaelis-Menten kinetic for GST-CK1ε, K_m_(ATP) = 15.36 µM. K_m_: Michaelis constant for ATP. Michaelis-Menten enzyme kinetic was applied to determine K_m_. K_m_(ATP) for GST-CK1δ was established by Roth *et al.* (Figure 7) and the data is licensed under a Creative Commons Attribution 4.0 International License (CC BY 4.0) (Roth et al., 2021).

Since all SMIs used in this study are ATP-competitive inhibitors, the ATP-concentration in the reaction mix for *in vitro* kinase assays has significant influence on the inhibitor assessment. The ATP-binding properties can differ between the CK1 isoforms, thus defining one standard ATP-concentration does not lead to comparable results. Therefore, kinase reactions with the previously established values for the ideal kinase concentration and reaction time were performed using different ATP-concentrations to determine the kinase-specific K_m_ of ATP (K_m_ (ATP)) by evaluating the resulting Michaelis-Menten kinetics (Figure 5). Additionally, by applying the ATP-concentration corresponding to the determined K_m_(ATP), the Cheng-Prusoff equation for calculating the kinase-specific K_i_ of an inhibitor gets beneficially simplified.

In Supplementary Table S5 all established standard conditions are summarized. All following *in vitro* kinase assays were performed pursuant to the determined values.

### 3.3 Small CK1δ specific inhibitors influence embryonic development of zebrafish

**FIGURE 6:**
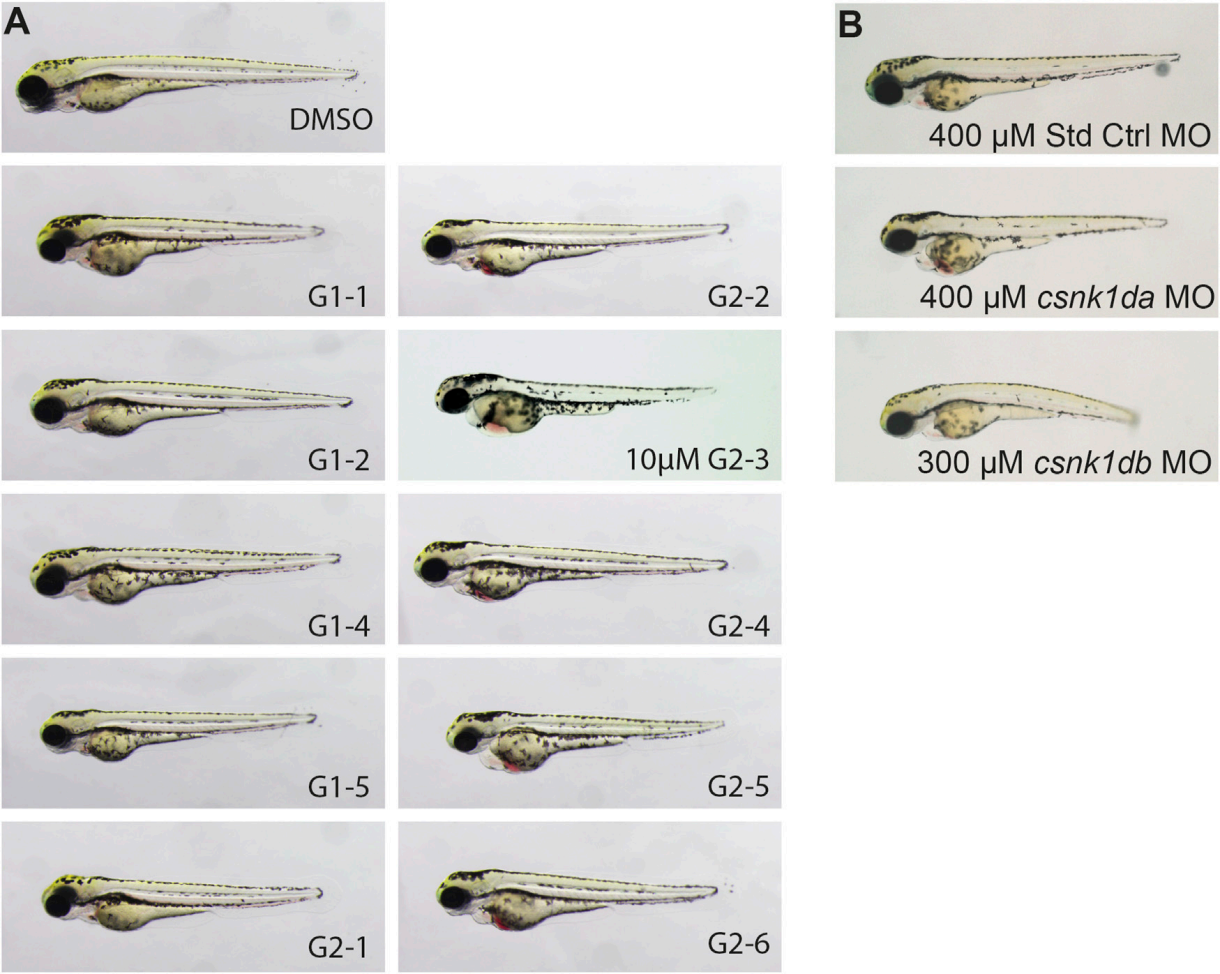
Representative pictures of SMI treated 72 hpf zebrafish embryos after 48 h of treatment and morpholino injected 72 hpf embryos. Treatment with G1-2, G1-3, G1-4 and G2-5 had no effect on zebrafish development or heart function. Treatment with 20 µM of G1-1 and G1-5 had a weak effect on heart development and function with a mild bradycardia and reduced heart rate. Treatment with G2-1, G2-2, G2-4, G2-6, and 10 µM G2-3 resulted in a strong cardiac phenotype, with blood congestion, a strong bradycardia and partially total loss of heart beat **(A)**. Injection of 400 µM Standard Control morpholino (Std Ctrl MO) had no phenotypic influence, injection of 400 µM *csnk1da* and 300 µM *csnk1db* morpholino lead to a reduced heart function resulting in blood congestion **(B)** (*n* = 10 for each compound, *n* = 30 for each morpholino).

After establishing *in silico* and *in vitro* that zebrafish CK1δ is functionally comparable to its human orthologue, the effect of CK1δ specific SMIs on zebrafish development was analyzed *in vivo*. Based on their ability to inhibit CK1δ *in vitro*, a set of SMIs was selected. Embryos were dechorionated at 24 hpf and treated with 20 µM of the respective inhibitor (Figure 6A).

G1-2, G1-3, G1-4 and G2-5 did not show a phenotypic effect on the developing zebrafish embryo (Figure 6A). G1-1 and G1-5 had mild effects on embryonic development and cardiac function, with G1-1 inducing a mild bradycardia and G1-5 a curved tail. G2-1, G2-2, G2-3, G2-4 and G2-6 had severe effects on the embryonic development, with strong bradycardia and blood congestion. G2-3 treated embryos were necrotic after 24 h of treatment, but even treatment with 10 µM had a severe effect on zebrafish embryos. Supplementary Table S6 summarizes the phenotypic changes in zebrafish embryos after treatment with the different CK1δ specific SMIs. For comparison, a CK1δ-knockdown was performed using morpholinos targeting the translational start site of zebrafish *csnk1da* (CK1δA) and *csnk1db* (CK1δB) that resulted in corresponding phenotypic abnormalities, like cardiac malfunction for both morpholinos, blood congestion in the case of *csnk1da* and a slightly curved body axis in the case of *csnk1db* (Figure 6B).

Based on these results, the five inhibitors that caused the most severe impairment on zebrafish embryogenesis (G2-1, G2-2, G2-3, G2-4 and G2-6) were evaluated further *in vitro* and *in vivo*.

### 3.4 *In vitro* comparison of human and zebrafish CK1 inhibition reveals no significant differences in inhibition by SMIs

**FIGURE 7:**
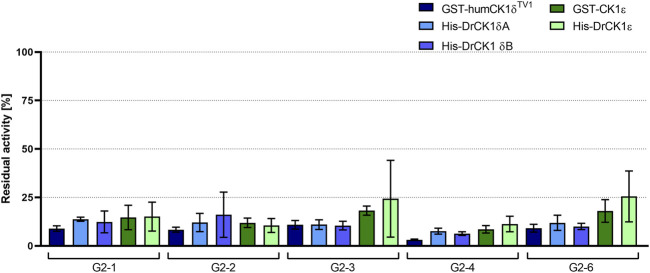
Initial screening of human as well as zebrafish CK1δ and ε with selected inhibitors. The selected inhibitors G2-1, G2-2, G2-3 ***,*** G2-4 and G2-6 were screened at a concentration of 20 µM in *in vitro* kinase assays with GST-humCK1δ^TV1^, GST-CK1ε, His-DrCK1δA, His-DrCK1δB and His-DrCK1ε. The residual kinase activity [%] was determined by measuring the radioactively labelled **γ**-phosphate incorporation into the substrate α-casein by Cherenkov counting. The results of the inhibitor-treated samples were normalized against the DMSO control, plotted against the respective inhibitor and are presented ±standard deviation.

The compounds G2-1, G2-2, G2-3, G2-4 and G2-6 were evaluated regarding their inhibitory potential and selectivity on human *versus* zebrafish CK1δ and ε *in vitro*. In compliance with the established standard conditions the kinase assays were performed in the presence of 20 µM of the respective inhibitor (Figure 7).

The percentage of residual kinase activity for each of the five screened compounds is in the same order of magnitude for the human CK1 isoforms δ and ε, compared to their zebrafish counterparts. Supplementary Table S7 summarizes the mean residual activities of the human and zebrafish CK1 isoforms δ and ε after treatment with the respective inhibitor. Tendentially, the selected compounds seemed to have a slightly stronger inhibitory effect on the CK1δ variants than on CK1ε.

### 3.5 Effects of CK1-inhibition on zebrafish embryogenesis is dose-dependent

**FIGURE 8:**
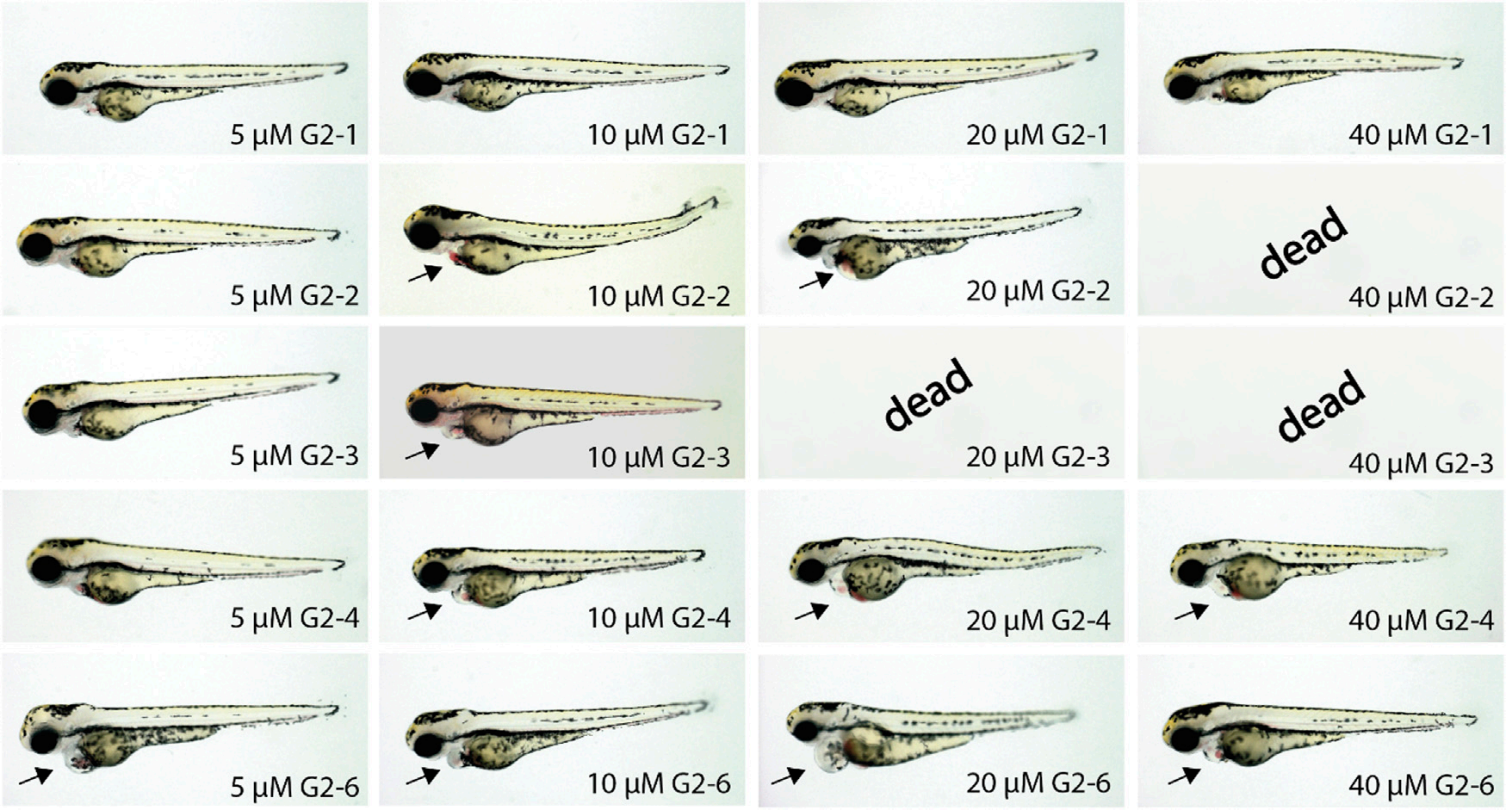
Representative images of SMI-treated zebrafish embryos after 48 h of incubation. Treatment of zebrafish embryos with G2-1, G2-4 and G2-6 showed increasingly severe cardiac malfunctions with increasing compound concentrations. The SMIs G2-2 and G2-3 induced necrosis at concentrations of 40 or 20 μM, respectively. Arrow indicates blood congestion and resulting cardiac edema (n = 20 for each compound).

Next, the five compounds G2-1, G2-2, G2-3, G2-4 and G2-6 were characterized regarding their influence on zebrafish embryogenesis after treatment with different doses of SMIs (5, 10, 20 and 40 µM) (Figure 8).

Inhibitor treated zebrafish embryos developed a similar phenotype but with differences in the severity of the impairment. At a concentration of 5 µM G2-1 did not show any phenotype, whereas G2-4 induced a mild phenotype with a minor blood congestion while G2-2, G2-3 and G2-6 developed a cardiac phenotype, with bradycardia and blood congestion, resulting in cardiac edema in the case of G2-6. Treatment with 10 µM of G2-6 resulted in bradycardia. G2-4 also showed a weakly beating ventricle and a pericardial edema. Treatment with G2-2 and G2-6 resulted in blood congestion and pericardial edema, whereas treatment with G2-3 led to blood congestion, reduced blood flow, a pericardial edema, and some of the embryos were starting to get necrotic. Treating embryos with 20 µM of the respective inhibitor led to bradycardia for all of the tested inhibitors, treatment with G2-1, G2-2 and G2-6 also resulted in blood congestions and after treatment with G2-2, G2-4 and G2-6 a pericardial edema was observed. Additionally, the ventricle in G2-4-treated embryos was almost not beating. Embryos treated with 20 µM G2-3 were dead after 48 h of treatment. 40 μM G2-1, G2-4 and G2-6 induced bradycardia, a loss of ventricular beating and as a result pericardial edema. At a concentration of 40 µM G2-2 and G2-3 were highly toxic, and all treated embryos were dead after 48 h of treatment. Those two are thus the most potent inhibitors in this experiment.

### 3.6 The two IWP-based inhibitors G2-2 and G2-3 effectively inhibit zebrafish CK1δ *in silico*, *in vitro* and *in vivo*

As they had a strong effect *in vivo*, the two IWP-based inhibitors G2-2 and G2-3 were selected for *in silico* modelling of kinase-inhibitor interactions, to determine IC_50_- as well as K_i_-values and for a more detailed analysis of the heart phenotype.

**FIGURE 9:**
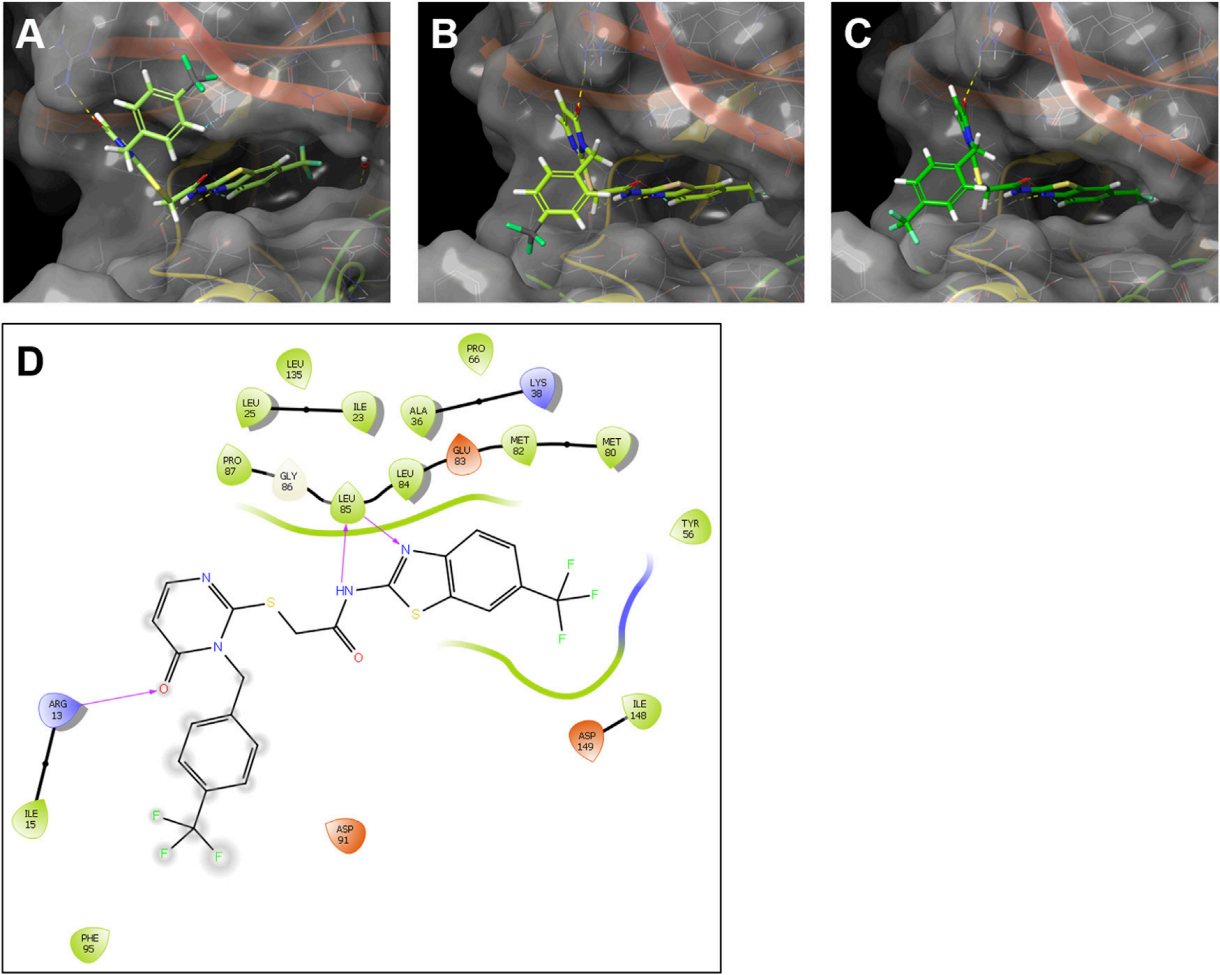
Modelling of the interaction of the SMI G2-2 with human CK1δ and the zebrafish CK1δ variants **(A, B)**. Ligand-protein models of G2-2 with human CK1δ (PDB code 5OKT) **(A)**, zebrafish CK1δA **(B)** and zebrafish CK1δB **(C)** were generated using Schrödinger Maestro. Hydrogen bonds between the inhibitor and the respective kinase are shown in dashed lines. **(D)** Exemplary 2D ligand interaction diagram of zebrafish CK1δA with G2-2. The hydrogen bonds are shown as purple arrows.

Although human and zebrafish CK1δ are highly homologous, especially in their kinase domain, there are differences in their respective amino acid sequences that can potentially influence the inhibitor binding properties. Therefore, the interaction of the SMIs G2-2 and G2-3 with the zebrafish CK1δ variants A and B was modelled and compared to the ligand-protein model of these inhibitors with human CK1δ. The potential interaction of the inhibitor with the different kinases was modelled using Schrödinger Maestro and the results for G2-2 are shown in Figure 9 and for G2-3 in Supplementary Figure S2.

Modelling analysis suggested highly comparable ligand-protein binding interactions for both, human and zebrafish CK1δ with the IWP-derivatives G2-2 and G2-3 (see Figure 9A–C and Supplementary Figure S2). In line with this notion, a 2D ligand interaction diagram (LID, see Figure 9D) predicts binding properties of compound G2-2 in zebrafish CK1δA that resonate with the binding model created by García Reyes et al. for the structurally similar benzimidazole-based inhibitor Bischof-5 in human CK1δ (García-Reyes et al., 2018).

**FIGURE 10:**
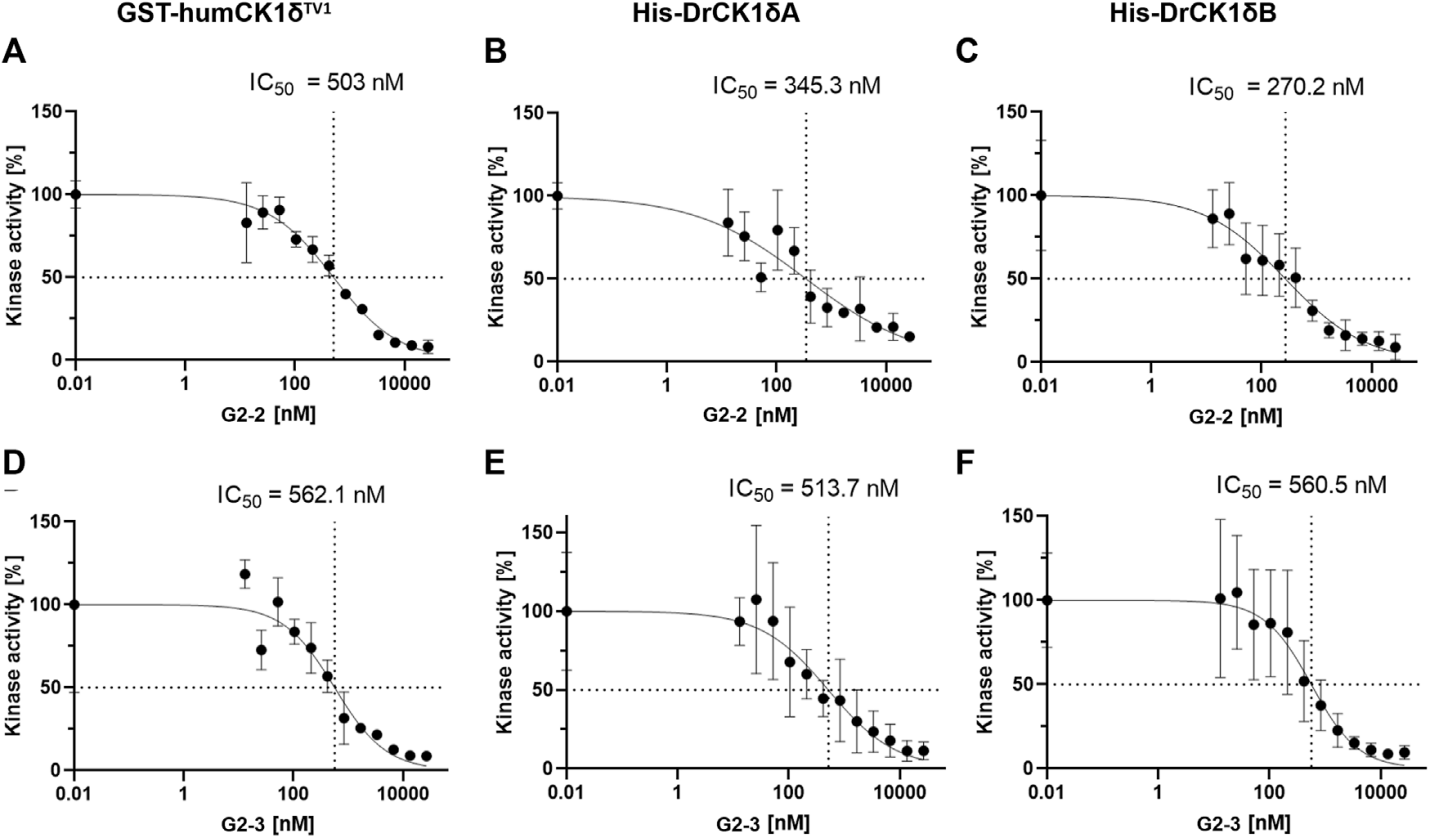
IC_50_-determination of G2-2 and G2-3 with GST-humCK1δ^TV1^, His-DrCK1δA and His-DrCK1δB. The IWP-derived inhibitors G2-2 and G2-3 were further assessed and IC_50_-values were determined. The transfer of radioactively labelled γ-phosphate to the substrate α-casein was measured by Cherenkov counting. The inhibitors were applied in different concentrations ranging from 13 to 26667 nM. The standardized kinase- and ATP-concentration as well as reaction time can be found in Supplementary Table S5. The kinase activity of the inhibitor-treated samples was normalized to the DMSO-controls. The results are shown as the mean value ± standard deviation. The experiments were conducted in technical triplicates. **(A)** IC_50_-determination of G2-2 with GST-humCK1δ^TV1^. **(B)** IC_50_-determination of G2-2 with His-DrCK1δA. **(C)** IC_50_-determination of G2-2 with His-DrCK1δB. IC_50_: 50% inhibitory concentration. **(D)** IC_50_-determination of G2-3 with GST-humCK1δ^TV1^. **(E)** IC_50_-determination of G2-3 with His-DrCK1δA. **(F)** IC_50_-determination of G2-3 with His-DrCK1δB. IC_50_: 50% inhibitory concentration.

Next, *in vitro* kinase assays were performed in absence and presence of the respective SMI at different concentrations (ranging from 13 to 26667 nM). The kinase activity of the inhibitor-treated samples was normalized to the DMSO-control and plotted against the inhibitor concentration (Figure 10).

The IC_50_-values of G2-2 for GST-humCK1δ^TV1^ (503 nM), His-DrCK1δA (345.3 nM) and His-DrCK1δB (270.2 nM) were indeed of the same order of magnitude but nevertheless there were differences in the 50% inhibitory concentration between the human CK1 and the zebrafish CK1 variants. The IC_50_-values of His-DrCK1δA and δB are relatively similar while the IC_50_ for GST-humCK1δ^TV1^ is 157.7 and 232.8 nM, respectively, higher. For G2-3 the similarity of the IC_50_-values was remarkable with 50% inhibitory concentrations of 562.1 nM for GST-humCK1δ^TV1^, 513.7 nM for His-DrCK1δA and 560.5 nM for His-DrCK1δB (summarized in Supplementary Table S8).

**FIGURE 11:**
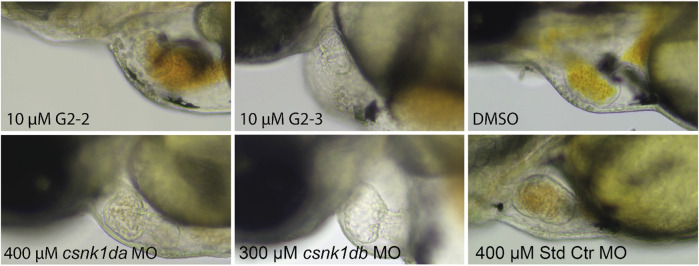
Representative pictures of hearts of inhibitor-treated and *csnk1d* MO injected embryos. Embryos were either treated with DMSO, 10 µM G2-2 or 10 µM G2-3 for 48 h from 24 hpf, or injected with *csnk1da, csnk1db* or control Morpholino (Std Ctr). DMSO and Std Ctr MO had wildtype heart morphology and function. Treatment with 10 µM G2-2 and G2-3 as well as injection of 400 µM *csnk1da* or 300 µM *csnk1db* Morpholino resulted in reduced heart function (*n* = 30 for each compound and morpholino).

As G2-2 and G2-3 were lethal to embryos at concentrations of 20 and 40 μM, experiments were performed at a concentration of 10 µM. Hearts of inhibitor treated embryos did not pump blood. This was also observed in the *csnk1da* and *csnk1db* morpholino injected embryos in a slightly weakened effect (Supplementary Movies S1–6). Figure 11 shows stand still pictures of the hearts from 72 hpf embryos. A blood congestion can be observed in the developing hearts.

## 4 Discussion

The zebrafish animal model has a variety of advantageous properties for studying vertebrate development and disease, and many mechanisms of physiological and pathological processes have been uncovered using the tropical fish (Penberthy et al., 2002). Recently, zebrafish gained the attention of precision oncology, where they could potentially be used to study individual disease progression and drug susceptibility (Cheng et al., 2011; Fazio et al., 2020; Hason et al., 2022). Zebrafish embryos, in accordance with the 3R principles, are increasingly used for drug screens in the early phase of drug development to obtain initial information on pharmacokinetic parameters, efficacy and toxicity of drugs before more advanced experiments are then performed in mouse models (Strähle et al., 2012). In addition, the high number of progenies makes it easy to compare different conditions (SMI, concentration, incubation time). As a result, more and more drugs entering preclinical and clinical trials are being tested in zebrafish (Patton et al., 2021). Replacement of the mouse model is of particular interest for studies of CK1 inhibition, as experiments in the past often had to be terminated due to significant adverse effects depending on the mouse strain. In C57/BL6 mice, oral administration of benzimidazole derivatives resulted in severe intestinal bleeding, often leading to death. By switching to a model with limited pain sensitivity, unnecessary animal distress and suffering can be avoided and rodent testing can be significantly reduced. Nevertheless, the effects of the inhibitors on the subcellular localization of CK1δ and its interaction with cellular proteins are difficult to study in zebrafish models, due to the low conservation of the responsible C-terminus, thus they need to be addressed in mouse models.

CK1 specific SMIs have a high potential for new therapeutic concepts in those diseases where dysregulation of CK1 contributes to the development and progression of disease states, including cancer. Therefore, the present study aimed to test whether zebrafish embryos can be used to screen newly developed CK1 isoform-specific inhibitors. As a readout of the efficacy of the new compounds tested, we aimed to use developmental abnormalities occurring in early embryogenesis, since the use of CK1 specific morpholinos lead to abnormal development, especially of the cardiovascular system. Before we performed the screening in zebrafish embryos of the selected IWP-derivatives, developed to specifically inhibit CK1δ in humans, the kinetic parameters of zebrafish CK1 isoforms δA, δB and ε were first determined under standard conditions, and compared to those of human CK1δ and ε. Determination of the kinetic parameters of zebrafish CK1 isoforms δA and δB under standard conditions revealed only minor differences in K_m_-values between DrCK1δA and B and humCK1δ^TV1^ as well as between DrCK1ε and humCK1ε.

Evaluation of the treatment effects of selected SMIs on zebrafish CK1δ isoforms and human CK1δ *in vitro* revealed that the zebrafish CK1 isoforms δA and δB do not differ from human CK1δ in the potential to be inhibited by G2-1, G2-2, G2-3, G2-4, and G2-6 under standard conditions (Supplementary Table S7).

Since *in silico* modelling of the interaction of SMIs with their target is a valuable tool to predict the inhibitory potential of a compound on a kinase of interest (Lyne et al., 2006; Abdelbaky et al., 2021; Ma et al., 2021), we performed a modelling analysis of the interaction of the compounds G2-2 and G2-3 with CK1δA, CK1δB and human CK1δ. A highly comparable ligand-protein binding was revealed suggesting a similar influence of ATP-competitive inhibitors on human and zebrafish CK1δ. Furthermore, the results were in line with the binding model for the structurally similar benzimidazole-based inhibitor Bischof-5 in CK1δ proposed by García-Reyes et al. (García-Reyes et al., 2018). The selected compounds did have a slightly weaker effect on the CK1ε variants, a tendency that was also observed by Liu and coworkers for their compounds 21 and 22 as well as by García-Reyes and coworkers for their compounds 17 and 20 (García-Reyes et al., 2018; Liu et al., 2019).

Determination of IC_50_- and K_i_-values for His-DrCK1δA, His-DrCK1δB and GST-humCK1δ^TV1^ with G2-2 and G2-3 (Liu et al., 2019) revealed that they all were in the same three-digit nanomolar range. In line with these results are previous observations. For example, human and zebrafish PKD2 were both able to phosphorylate HDAC5 *in vitro* (Just et al., 2011). Additionally, it was shown that the inhibitor H89 has a comparable, but slightly lower IC_50_ value for human PKCα (31.2 µM) than for zebrafish PKCα (49.8 µM) (Qiao et al., 2021).

Based on the *in vitro* inhibitor studies, a dose-dependent effect study of inhibitors G2-1, G2-2, G2-3, G2-4, and G2-6 was performed in the zebrafish model for 48 h to assess the effects on early zebrafish embryonic development. Except from G2-1, all other compounds induced a mild phenotype with minor blood congestion (G2-4) or a cardiac phenotype (G2-2, G2-3 and G2-6) already at a concentration of 5 µM. The impact of the inhibitor treatment increased in a dose-dependent manner and the embryos developed severe bradycardia, blood congestion, pericardial edema and loss of ventricular beating. A dose-dependent increase in toxicity was expected and has been described for other SMIs as well (Cassar et al., 2020; Wanting et al., 2023). G2-2 and G2-3 were demonstrated to be highly toxic and the most potent compounds from this set. Interestingly, the SMI G2-2 seemed to be more efficient in inhibiting CK1δ *in vitro* while the *in vivo* assessment revealed a heavier impact of G2-3 on embryogenesis. This is unexpected but not uncommon, as Lee and colleagues highlight that the standard conditions used for *in vitro* experiments are far from what can be expected *in vivo*, and therefore variation may occur, e.g., based on variable pharmacokinetics (Lee et al., 2019). Liu et al. reported slightly more inhibitory potential for G2-2 compared to G2-3 *in vitro* whereas G2-3 was able to reduce cell viability in three out of four tested colon cancer cell lines more efficiently, but still moderately, than G2-2 (Liu et al., 2019). The *in silico* analysis suggested highly comparable binding properties of the two IWP-based compounds for human and zebrafish CK1δ but the differences in electronegativity and lipophilicity between G2-2 with a trifluoromethyl group instead of the fluoro-group of G2-3 could influence their reactivity or ability to pass cell membranes (see Supplementary Table S2). For the trifluoromethyl group of G2-2, a stronger -I-effect would be expected which could lead to increased lipophilicity (Kubinyi, 1986). From this premise, it is interesting that G2-2 was slightly less efficient than G2-3 regarding its *in vivo* inhibitory effects. The phenotypes correspond to the reported developmental impairments after an initial *in vivo* screening. Since the genetical knockdown of CK1δ using morpholinos did result in a comparable phenotype, it is most likely that in fact CK1δ inhibition caused the impairments and not unspecific adverse or off-target effects of the SMIs. Since CK1δ, as well as the other CK1 isoforms, were reported to be ubiquitously expressed during zebrafish embryogenesis (Albornoz et al., 2007), severe developmental impairment and reduced viability were expected upon CK1δ-downregulation. Albornoz et al. showed a prominent expression of CK1δA and δB in the embryos’ tegmentum and myelencephalon, structures that are responsible for cardiovascular control, explaining bradycardia and blood stasis as a result of inhibitor-treatment. Additionally, CK1δA was found to be expressed in the somites and tails of the embryos. Somites give rise to the vertebrae, the vertebrate column and the musculoskeletal system of the back, thereby the tail malformation upon inhibition of CK1δ could potentially be explained (Albornoz et al., 2007). The severe impairment of zebrafish embryogenesis also resonates with previous findings in mice, where CK1δ knockout results in underdeveloped pups and perinatal death (Etchegaray et al., 2009). Since the zebrafish embryos were treated with high doses between 5 and 20 µM inhibitor, the effects of lower SMI-concentrations should be evaluated in follow-up experiments to determine an inhibitor-concentration that can potentially interfere with tumor growth in cancer models while not inhibiting the activity of CK1δ in healthy cells to a point of severe damage and distinctly reduced viability.

In summary, in this study, we demonstrated that treatment of zebrafish with CK1δ-specific inhibitors can induce phenotypic systemic effects during embryogenesis. This demonstrates that the zebrafish model can be used for early drug screening of newly developed CK1 isoform-specific compounds.

## Data Availability

The original contributions presented in the study are included in the article/Supplementary Material, further inquiries can be directed to the corresponding author.
