# Supplementary material for: Zebrafish as model system for the biological characterization of CK1 inhibitors
Source: Front Pharmacol. 2023 Sep 11;14:1245246. doi: 10.3389/fphar.2023.1245246 (PMC10518421; doi:10.3389/fphar.2023.1245246)
Supplement: Supplementary file 2 [file Table1.DOCX]

**Supplementary Table 1:** Primer used for amplification and generation of complementary overhangs for subsequent ligation of the Dr csnk1 DNA fragments with the pET-28a(+) vector.

| **Primer name** | **Sequence** |
| --- | --- |
| Dr_Csnk1dA_fwd | 5’-agcaaatgggtcgcggatccatggaattgagagtaggaaaccg-3’ |
| Dr_Csnk1dA_rev | 5’-tggtggtggtggtgctcgagtcatcgaggtacggcagac-3’ |
| Dr_Csnk1dB_fwd | 5’-agcaaatgggtcgcggatccatggagctacgagttggaaac-3’ |
| Dr_Csnk1dB_rev | 5’-tggtggtggtggtgctcgagctacttgccgtggtgatc-3’ |
| Dr_Csnk1e_fwd | 5’-agcaaatgggtcgcggatccatggagttgcgtgttggaag-3’ |
| Dr_Csnk1e_rev | 5’-tggtggtggtggtgctcgagtcatttccccatgtgttcg-3’ |
