# Supplementary material for: Zebrafish as model system for the biological characterization of CK1 inhibitors
Source: Front Pharmacol. 2023 Sep 11;14:1245246. doi: 10.3389/fphar.2023.1245246 (PMC10518421; doi:10.3389/fphar.2023.1245246)
Supplement: Supplementary file 3 [file Table8.DOCX]

**Supplementary Table 8: Overview over the determined IC_50_-values as well as the resulting K_i_-values.** IC_50_-values of the inhibitors G2-2 and G2-3 were determined in in vitro kinase assays with GST-humCK1δ^TV1^_,_ His-DrCK1δA and His-DrCK1δB. IC_50_: 50 % inhibitory concentration, K_i_: inhibitor constant.

| **Kinase** | **Inhibitor** | **IC_50_ [nM]** | **K_i_ [nM]** |
| --- | --- | --- | --- |
| GST-humCK1δ^TV1^ | G2-2 | 503 | 251.5 |
|  | G2-3 | 562.1 | 281.05 |
| His-DrCK1δA | G2-2 | 345.3 | 172.65 |
|  | G2-3 | 513.7 | 256.85 |
| His-DrCK1δB | G2-2 | 270.2 | 135.1 |
|  | G2-3 | 560.5 | 280.25 |
