# Supplementary figures and images for: Zebrafish as model system for the biological characterization of CK1 inhibitors
Source: Front Pharmacol. 2023 Sep 11;14:1245246. doi: 10.3389/fphar.2023.1245246 (PMC10518421; doi:10.3389/fphar.2023.1245246)

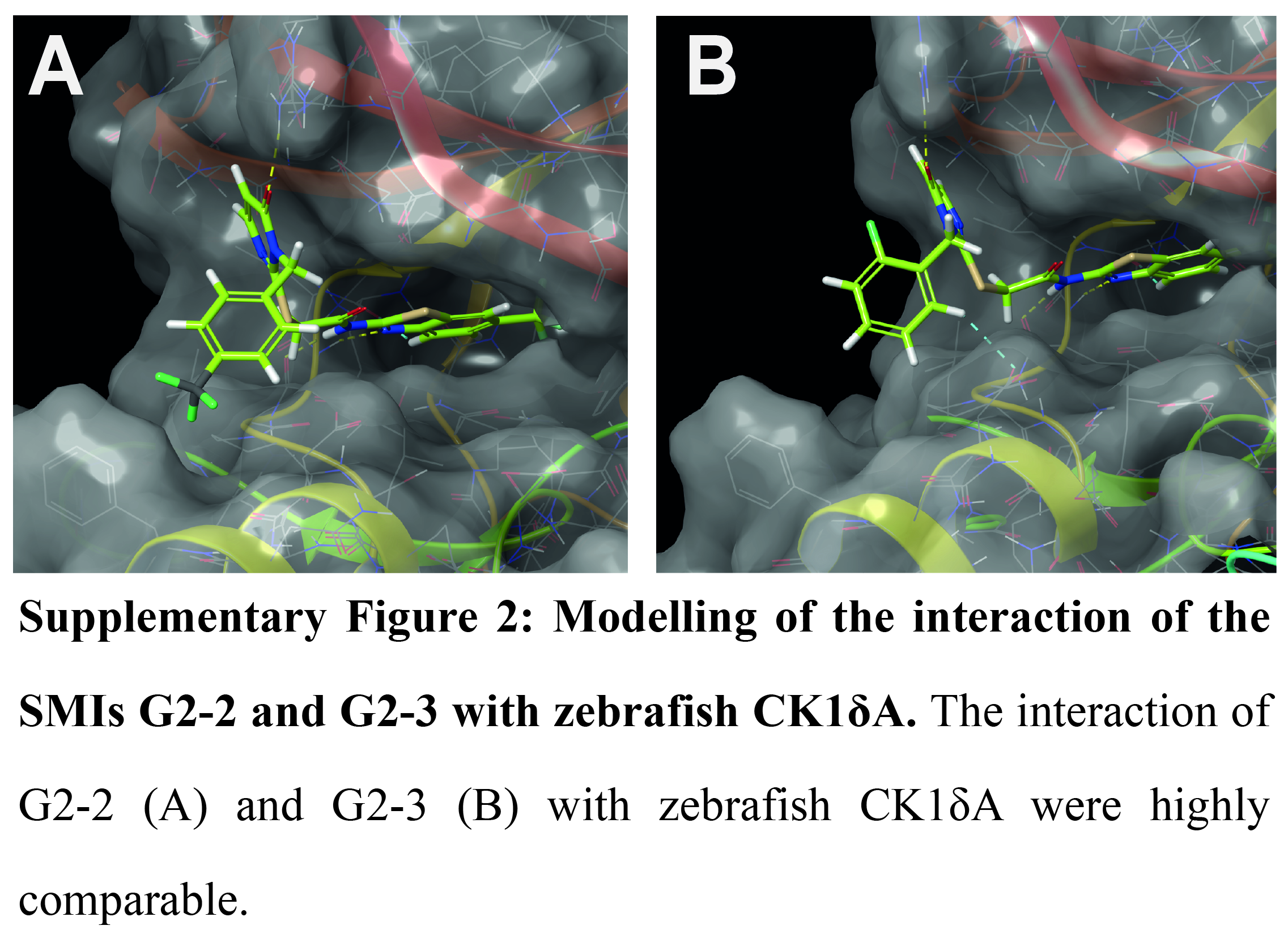

Supplement: Supplementary file 4 [file Image2.TIF]

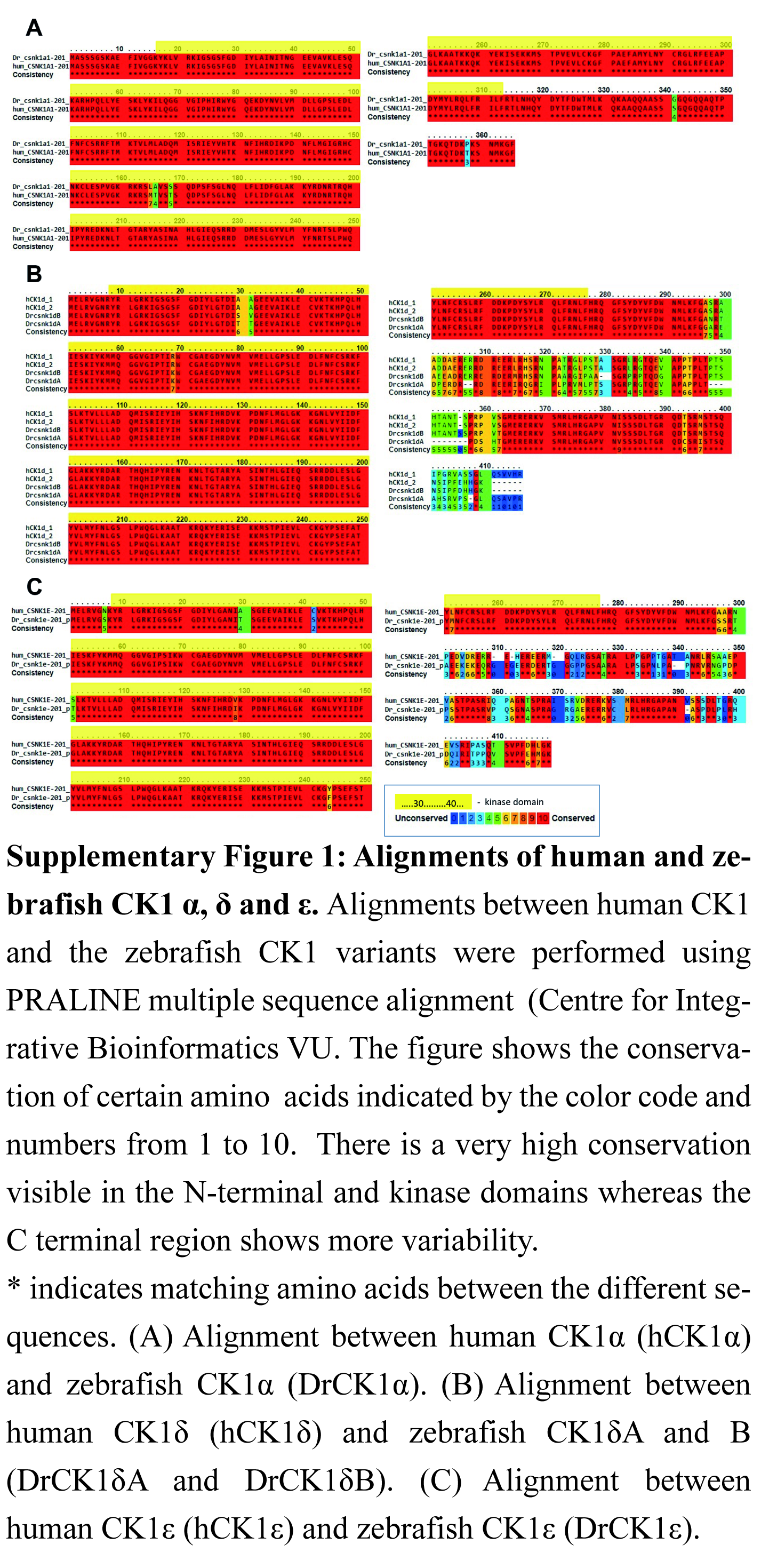

Supplement: Supplementary file 6 [file Image1.TIF]
