# Supplementary material for: Zebrafish as model system for the biological characterization of CK1 inhibitors
Source: Front Pharmacol. 2023 Sep 11;14:1245246. doi: 10.3389/fphar.2023.1245246 (PMC10518421; doi:10.3389/fphar.2023.1245246)
Supplement: Supplementary file 5 [file Table7.DOCX]

**Supplementary Table 7: Overview over the mean residual kinase activities ± SD [%] after treatment with the compounds G2-1, G2-2, G2-3, G2-4 and G2-6.**

|  | **GST-humCK1δ^TV1^** | **HisDrCK1δA** | **HisDrCK1δB** | **GST-CK1ε** | **His-DrCK1ε** |
| --- | --- | --- | --- | --- | --- |
| **G2-1** | 8.9 ± 1.2 % | 13.8 ± 0.9 % | 12.4 ± 4.6 % | 14.7 ± 5.2 % | 15.1 ± 6.1 % |
| **G2-2** | 8.4 ± 1.1 % | 12.1 ± 3.9 % | 16.1 ± 9.5 % | 11.9 ± 2.0 % | 10.5 ± 3.0 % |
| **G2-3** | 10.9 ± 1.8 % | 11.0 ± 2.1 % | 10.5 ± 1.8 % | 18.2 ± 1.9 % | 24.3 ± 16.2 % |
| **G2-4** | 3.2 ± 0.3 % | 7.6 ± 1.2% | 6.3 ± 0.8 % | 8.6 ± 1.6 % | 11.3 ± 3.3 % |
| **G2-6** | 9.1 ± 1.6 % | 11.9 ± 3.2 % | 10.0 ± 1.3 % | 18.0 ± 4.8 % | 25.5 ± 10.7% |
