# Supplementary material for: Zebrafish as model system for the biological characterization of CK1 inhibitors
Source: Front Pharmacol. 2023 Sep 11;14:1245246. doi: 10.3389/fphar.2023.1245246 (PMC10518421; doi:10.3389/fphar.2023.1245246)
Supplement: Supplementary file 7 [file Table2.DOCX]

**Supplementary Table 2:** Overview of all small molecule inhibitors used in this study.

| **Inhibitor name** | **Structure** | **M_W_ [g/mol]** | **IC_50_ values** | **Reference** |
| --- | --- | --- | --- | --- |
| G1-1 |  | 520.57 | CK1δ: 660 nM  CK1ε: 4.120 nM | (García-Reyes et al., 2018) |
| G1-2 |  | 594.65 | CK1δ: 760 nM  CK1ε: 1230 nM | (García-Reyes et al., 2018) |
| G1-3 |  | 602.59 | CK1δ: 330 nM  CK1ε: nd | (García-Reyes et al., 2018) |
| G1-4 |  | 594.65 | CK1δ (rat): 380 nM  ^M28F^CK1δ (rat): 160 nM  ^wt^CK1ε: 3200 nM | * |
| G1-5 |  | 486.55 | CK1δ (rat): 160 nM  CK1δ (TV1): 380 nM  CK1δ (TV2): 510 nM  ^M28F^CK1δ (rat): 120 nM  ^wt^CK1ε: 780 nM | * |
| G2-1 |  | 522.52 | CK1δ: 1.724 nM  CK1ε: nd | (Liu et al., 2019) |
| G2-2 |  | 544.49 | CK1δ: 120 nM  CK1ε: 926 nM* | (Liu et al., 2019) |
| G2-3 |  | 494.48 | CK1δ: 240 nM  CK1ε: 1.144 nM* | (Liu et al., 2019) |
| G2-4 |  | 492.49 | CK1δ (TV1): 970 nM  ^wt^CK1ε: nd | * |
| G2-5 |  | 492.49 | CK1δ (TV1): 840 nM  ^wt^CK1ε: nd | * |
| G2-6 |  | 492.49 | CK1δ (TV1): 1400 nM  ^wt^CK1ε: nd | * |

*: Not published, nd: not determined
