# Supplementary material for: Zebrafish as model system for the biological characterization of CK1 inhibitors
Source: Front Pharmacol. 2023 Sep 11;14:1245246. doi: 10.3389/fphar.2023.1245246 (PMC10518421; doi:10.3389/fphar.2023.1245246)
Supplement: Supplementary file 8 [file Table5.DOCX]

**Supplementary Table 5: Summary of all established standard conditions for *in vitro* kinase assays.** Standard conditions for GST-humCK1δ^TV1^ were previously established. Standard conditions for GST-CK1δ waere established by Roth *et al.* and the data is licensed under a Creative Commons Attribution 4.0 International License (CC BY 4.0) (Roth et al., 2021).

|  | **Applied kinase con-centration [nM]** | **Reaction time [min]** | **K_m_(ATP) [µM]** |
| --- | --- | --- | --- |
| **His-DrCK1δA** | 70 | 5 | 11.97 |
| **His-DrCK1δB** | 33 | 10 | 5.31 |
| **His-DrCK1ε** | 7 | 15 | 14.45 |
| **GST-humCK1δ^TV1^** * | 7* | 10* | 6.00* |
| **GST-humCK1ε** | 70 | 10 | 15.36 |

* Previously established by Roth et al. (Roth et al., 2021)
