# Supplementary material for: Zebrafish as model system for the biological characterization of CK1 inhibitors
Source: Front Pharmacol. 2023 Sep 11;14:1245246. doi: 10.3389/fphar.2023.1245246 (PMC10518421; doi:10.3389/fphar.2023.1245246)
Supplement: Supplementary file 9 [file Table3.DOCX]

**Supplementary Table 3: Summary of the determined kinase concentrations and the specific activities of the zebrafish CK1 isoforms.** 1 unit (U) is defined as 1 pmol phosphate that is transferred to the substrate in 1 minute at 30 °C and in a reaction volume of 15 µL. The kinase activity is specified in U per pmol kinase ± standard deviation.

| **Zebrafish kinase** | **Ideal concentration [nM]** | **Activity [U/pmol]** |
| --- | --- | --- |
| His-DrCK1δA | 70 | 0.13 ± 0.01 |
| His-DrCK1δB | 33 | 0.67 ± 0.08 |
| His-DrCK1ε | 7 | 1.46 ± 0.40 |
