# Supplementary material for: Zebrafish as model system for the biological characterization of CK1 inhibitors
Source: Front Pharmacol. 2023 Sep 11;14:1245246. doi: 10.3389/fphar.2023.1245246 (PMC10518421; doi:10.3389/fphar.2023.1245246)
Supplement: Supplementary file 11 [file Table4.DOCX]

**Supplementary Table 4: Determination of the initial velocity region for His‑DrCK1δA, δB, ε and GST-CK1ε.** Linear regression was conducted to determine the maximal R^2^. Therefore, the analyzed data points of the product-over-time progression curve were reduced step by step. N: sample size; R^2^: coefficient of determination. Reaction time for GST-CK1δ was established by Roth et al. (Figure 4) and the data is licensed under a Creative Commons Attribution 4.0 International License (CC BY 4.0) (Roth et al., 2021).

|  |  | **R^2^** | | | | |
| --- | --- | --- | --- | --- | --- | --- |
| **Time range [min]** | **n** | **His-DrCK1δA** | **His-DrCK1δB** | **GST-CK1δ*** | **His-DrCK1ε** | **GST-CK1ε** |
| 0-60 | 8 | 0.94 | 0.94 | 0.92* | 0.79 | 0.96 |
| 0-45 | 7 | 0.90 | 0.95 | 0.97* | 0.78 | 0.95 |
| 0-30 | 6 | 0.94 | 0.87 | 0.96* | 0.82 | 0.90 |
| 0-15 | 5 | 0.96 | 0.99 | **0.99*** | **0.98** | 0.86 |
| 0-10 | 4 | 0.93 | **0.99** | 0.99* | 0.95 | **0.96** |
| 0-5 | 3 | **0.98** | - | - | - | - |
| **Reaction time** | | **5 min** | **10 min** | **10 min*** | **15 min** | **10 min** |

*Previously established by Roth *et al.* (Roth et al., 2021)
