# Supplementary material for: Zebrafish as model system for the biological characterization of CK1 inhibitors
Source: Front Pharmacol. 2023 Sep 11;14:1245246. doi: 10.3389/fphar.2023.1245246 (PMC10518421; doi:10.3389/fphar.2023.1245246)
Supplement: Supplementary file 13 [file Table6.DOCX]

**Supplementary Table 6: List of the inhibitors that were selected for the *in vivo* screening of zebrafish embryonic development.** The compounds were selected based on their ability to inhibit CK1δ in *in vitro* kinase assays. The initial IC_50_-values were determined prior to the establishment of the standardized *in vitro* kinase assay protocol. The Supplementary Table summarizes, whether the zebrafish embryos presented an abnormal phenotype upon inhibitor treatment. The inhibitors were added to the zebrafish medium in a concentration of 20 µM.

| **Inhibitor** | **Phenotypic changes (with severity) after treatment of zebrafish embryos with 20 µM inhibitor** | **Phenotypic changes** |
| --- | --- | --- |
| G1-1 (García-Reyes et al., 2018) | Yes (mild) | Mild bradycardia |
| G1-2 (García-Reyes et al. 2018) | No | None observed |
| G1-3 (García-Reyes et al. 2018) | No | None observed |
| G1-4 | No | None observed |
| G1-5 | Yes (mild) | Tail slightly curved |
| G2-1 (Liu et al., 2019) | Yes (severe) | Bradycardia, reduced to no blood circulation, blood congestion |
| G2-2 (Liu et al., 2019) | Yes (severe) | Bradycardia, blood congestion, barely blood circulation, pericardial edema |
| G2-3 (Liu et al., 2019) | Yes (necrotic, 10 µM: severe) | Bradycardia, pericardial edema, reduced blood flow, cardiac arrest, |
| G2-4 | Yes (severe) | Bradycardia, weak ventricle beat, barely blood circulation, no movement |
| G2-5 | No | None observed |
| G2-6 | Yes (severe) | Bradycardia, blood, congestion, slow to no heart beat |
